# Supplementary material for: Twister ribozymes as highly versatile expression platforms for artificial riboswitches
Source: Nat Commun. 2016 Sep 27;7:12834. doi: 10.1038/ncomms12834 (PMC5052635; doi:10.1038/ncomms12834)
Supplement: Supplementary Information — Supplementary Figures 1-25, Supplementary Tables 1-7, Supplementary Notes 1-7 and Supplementary References. [file ncomms12834-s1.pdf]

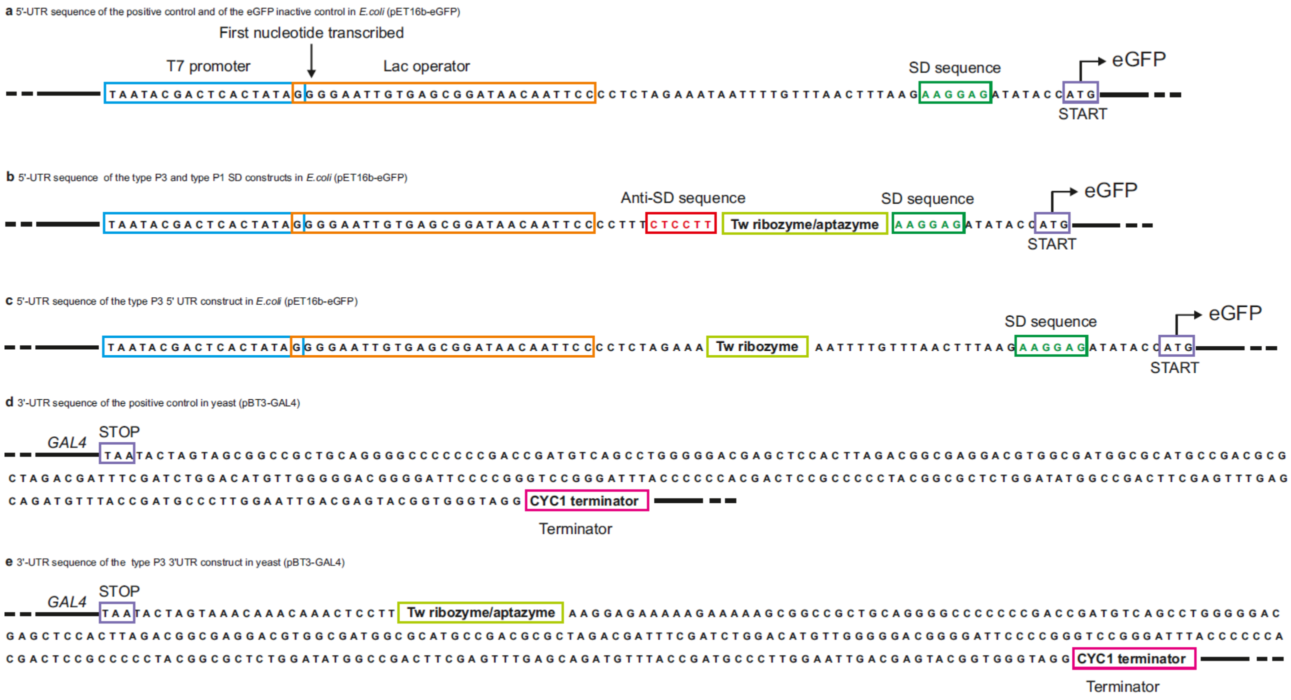

Supplementary Figure 1 | Sequence of the 5'-UTR of the eGFP gene in the constructs employed in *E. coli* and of the 3'-UTR of the *GAL4* gene in the constructs employed in yeast.
(**a**) Sequence of the 5'-UTR of the eGFP gene in the positive and in the eGFP inactive controls (**Figure 1b-c**). The T7 promoter is highlighted in blue, the Lac operator in orange, the Shine-Dalgarno sequence (SD) in green and the eGFP start codon in purple. (**b**) Sequence of the 5'-UTR of the eGFP gene in type P3 and type P1 SD constructs in *E.coli* (**Figure 1d-e**, **Figure 2a** and **Figure 3a**). Here the twister ribozyme acts as a molecular scaffold for the sequestration of SD sequence. The T7 promoter is highlighted in blue, the Lac operator in orange, the anti Shine-Dalgarno sequence (anti-SD) in red, the SD sequence in green and the eGFP start codon in purple. The site of insertion of the twister ribozyme or twister-based aptazyme is represented by a light green box. (**c**) Sequence of the 5'-UTR of the eGFP gene in type P3 5'UTR construct in *E.coli* (**Figure 1f**). Here the env-9 motif is inserted in the 5'-UTR, upstream of the SD sequence of the eGFP gene. The T7 promoter is highlighted in blue, the Lac operator in orange, the SD sequence in green and the eGFP start codon in purple. The site of insertion of the twister ribozyme is represented by a light green box. (**d**) Sequence of the 3'-UTR of the *GAL4* gene in the positive control in yeast. The *GAL4* stop codon is highlighted in purple and the CYC1 terminator in magenta. (**e**) Sequence of the 3'-UTR of the *GAL4* gene in the in type P3 3'UTR construct in yeast (**Figure 2b**). The *GAL4* stop codon in highlighted in purple and the CYC1 terminator in magenta. The site of insertion of the twister ribozyme or twister-based aptazyme is represented by a light green box.

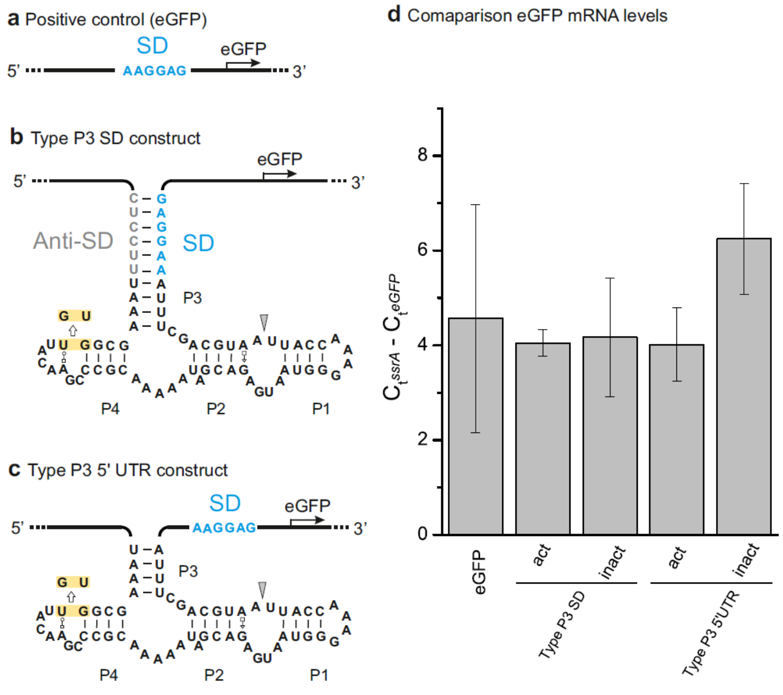

Supplementary Figure 2 | Effects of the presence of the twister catalytic motif in the 5'-UTR of the eGFP mRNA levels. The mRNA levels of five different constructs are compared. (**a**) The positive control. (**b**) The twister ribozyme acts as a molecular scaffold for the sequestration of Shine-Dalgarno sequence (SD) sequence. (**c**) The env-9 motif is inserted in the 5'-UTR, upstream of the SD of the eGFP gene. Cleavage-inactive constructs were generated by inverting two conserved nucleotides highlighted in yellow in (**b**) and (**c**). In (**d**) the ΔC_t values (calculated as C_t*ssrA* - C_t*eGFP*) are shown for each of the five constructs. The error bars represent standard deviation calculated on independent biological triplicates.

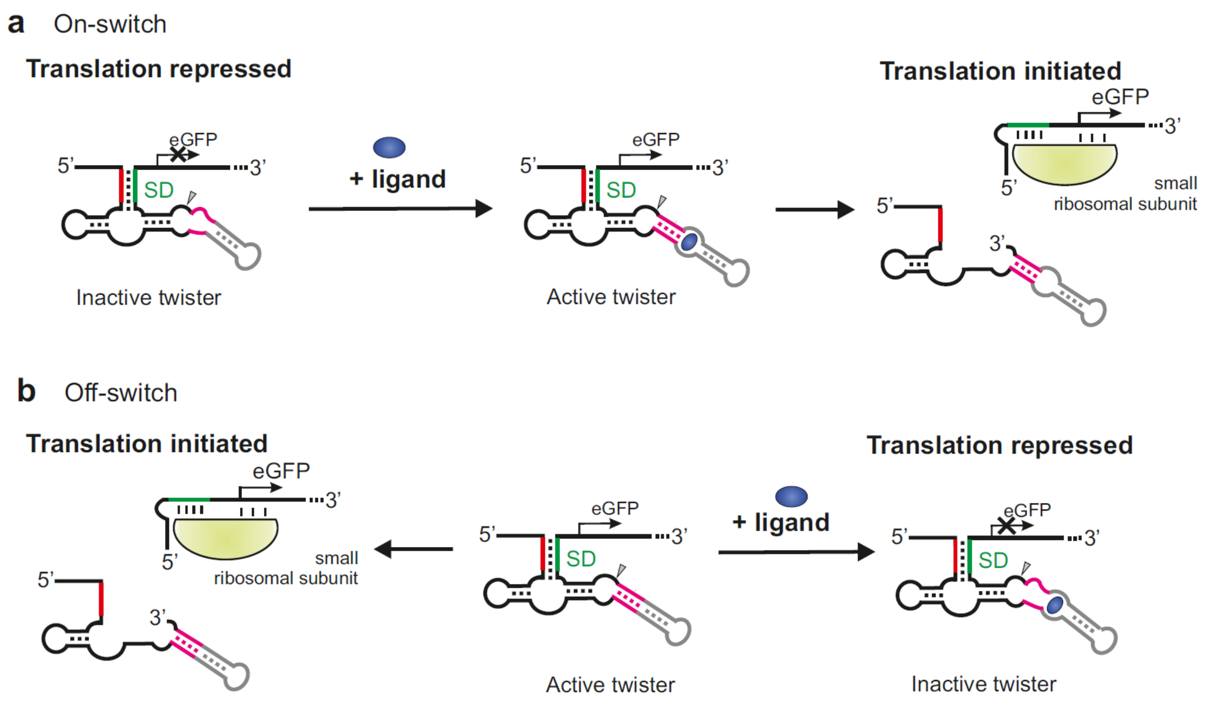

Supplementary Figure 3 | Mechanisms of twister-based on- and off-switches in *E. coli*. In our approach the twister ribozyme acts as a molecular scaffold for the sequestration of the Shine-Dalgarno sequence (SD). Aptamer domains can be attached to the ribozyme as exchangeable ligand-sensing domains. Addition of ligands to the growth medium changes the activity of the ligand-dependent self-cleaving ribozyme which in turn switches gene expression on (**a**) or off (**b**).

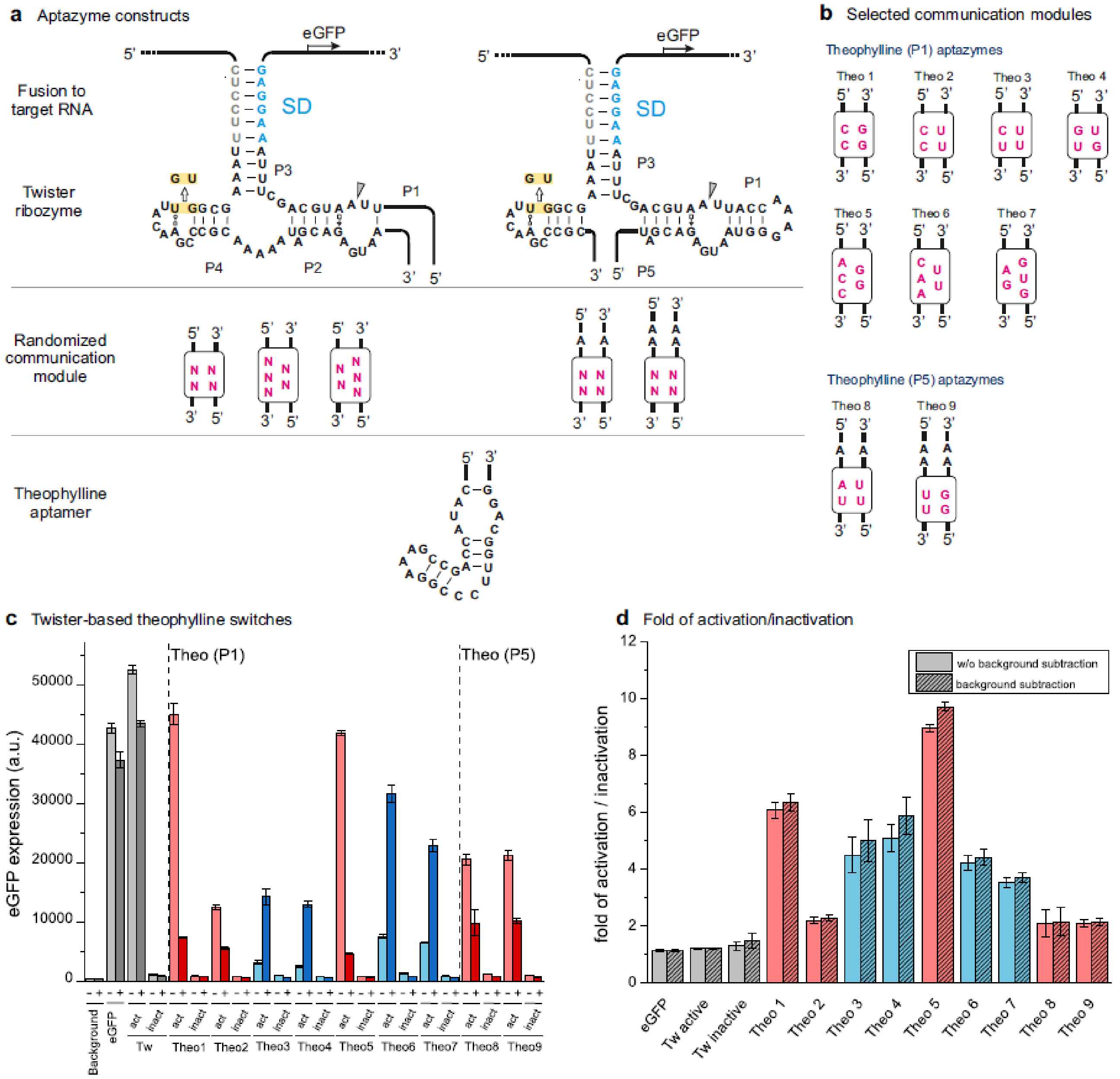

Supplementary Figure 4 | Design, sequences and performances of the theophylline twister-based riboswitches. (**a**) The theophylline aptamer was connected to the stem P1 using three different types of communication module: one symmetric containing four randomized nucleotides and two asymmetric containing five randomized nucleotides. In the P5 theophylline aptazymes, symmetric communication modules of four randomized nucleotides were inserted by replacing a variable number of adenosine residues in the accommodating loop. Cleavage-inactive control constructs were generated by inverting two conserved nucleotides highlighted in yellow. The cleavage site is indicated with a grey arrow. (**b**) The identified communication modules of the screened theophylline riboswitches. (**c**) Levels of eGFP expression of the selected theophylline clones in the absence (-) and in the presence (+) of 2.5 mM theophylline. The levels of reporter gene expression in the different conditions are represented by gray tone bars for the controls, red tone bars for the off-switches and blue tone bars for the on-switches. The error bars represent standard deviations calculated on independent biological triplicates (**d**) The performances (fold of activation or inactivation) of the on- and off-switches were calculated as the ratio of fluorescence of active divided by inactive expression state and are represented with blue (on-switches) and red (off-switches) bars respectively. The performances of the switches calculated without and with background subtraction (empty and striped bars respectively) are reported in the graph. The error bars represent standard deviations calculated on independent biological triplicates

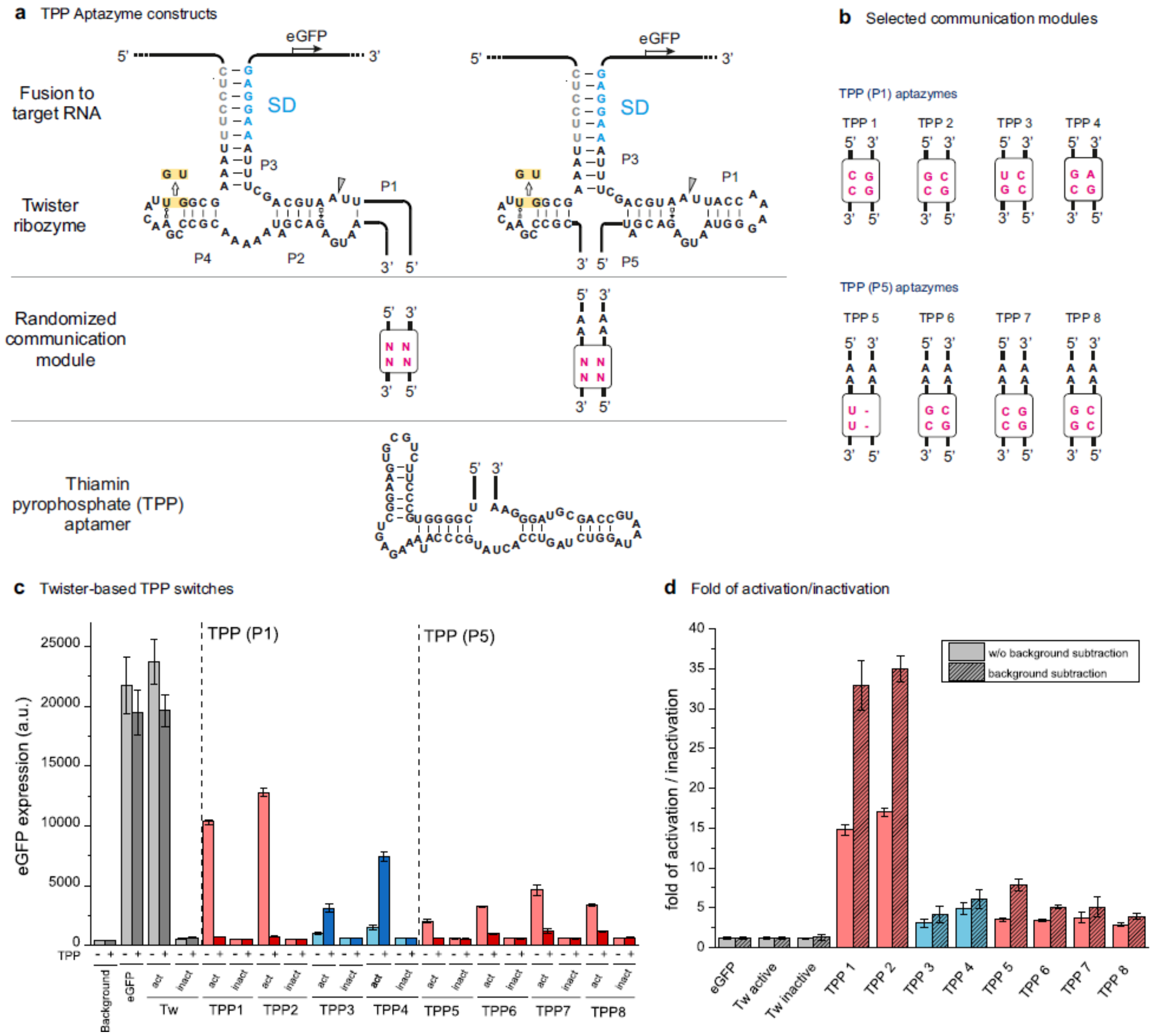

Supplementary Figure 5 | Design, sequences and performances of the TPP twister-based riboswitches. (**a**) The TPP P1 library was designed using a symmetric communication module of four randomized nucleotides. In the TPP P5 library, the communication module of four randomized nucleotides was inserted with two flanking adenosine residues on each side. Cleavage-inactive control constructs were generated by inverting two conserved nucleotides highlighted in yellow. The cleavage site is indicated with a grey arrow. (**b**) The identified communication modules of the screened TPP riboswitches. (**c**) Levels of eGFP expression of the selected TPP clones in the absence (-) and in the presence (+) of 1 mM thiamin. The levels of reporter gene expression in the different conditions are represented by gray tone bars for the controls, red tone bars for the off-switches and blue tone bars for the on-switches. The error bars represent standard deviations calculated on independent biological triplicates (**d**) The performances (fold of activation or inactivation) of the on- and off-switches were calculated as the ratio of fluorescence of active divided by inactive expression state and are represented with blue (on-switches) and red (off-switches) bars respectively. The performances of the switches calculated without and with background subtraction (empty and striped bars respectively) are reported in the graph. The error bars represent standard deviations calculated on independent biological triplicates

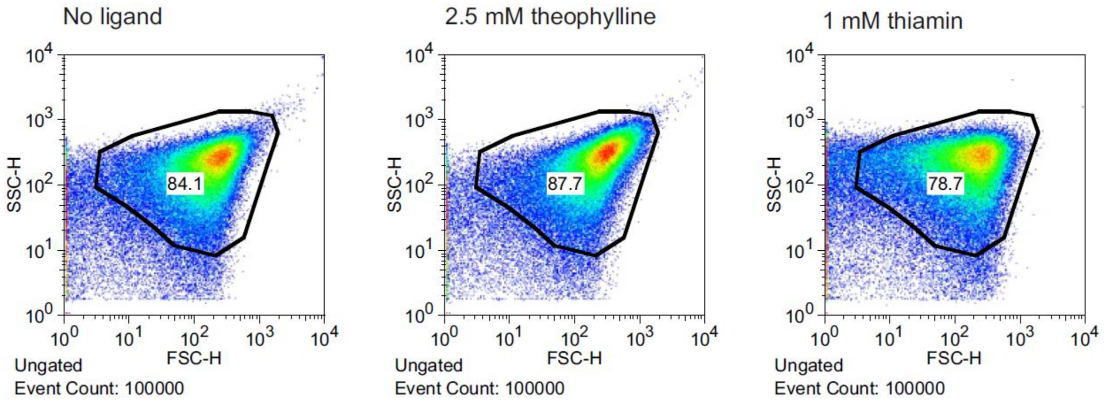

Supplementary Figure 6 | Gate used for the analysis of the Flow cytometry (FC) data. Forward and side scatter (FSC-H and SSC-H) plots of the eGFP control in three different culture conditions (no ligand, 2.5 mM theophylline and 1 mM thiamine). The gate employed for the analysis of the FC data is indicated with a black line. The percentages of events (cells) included in the gated population is shown.

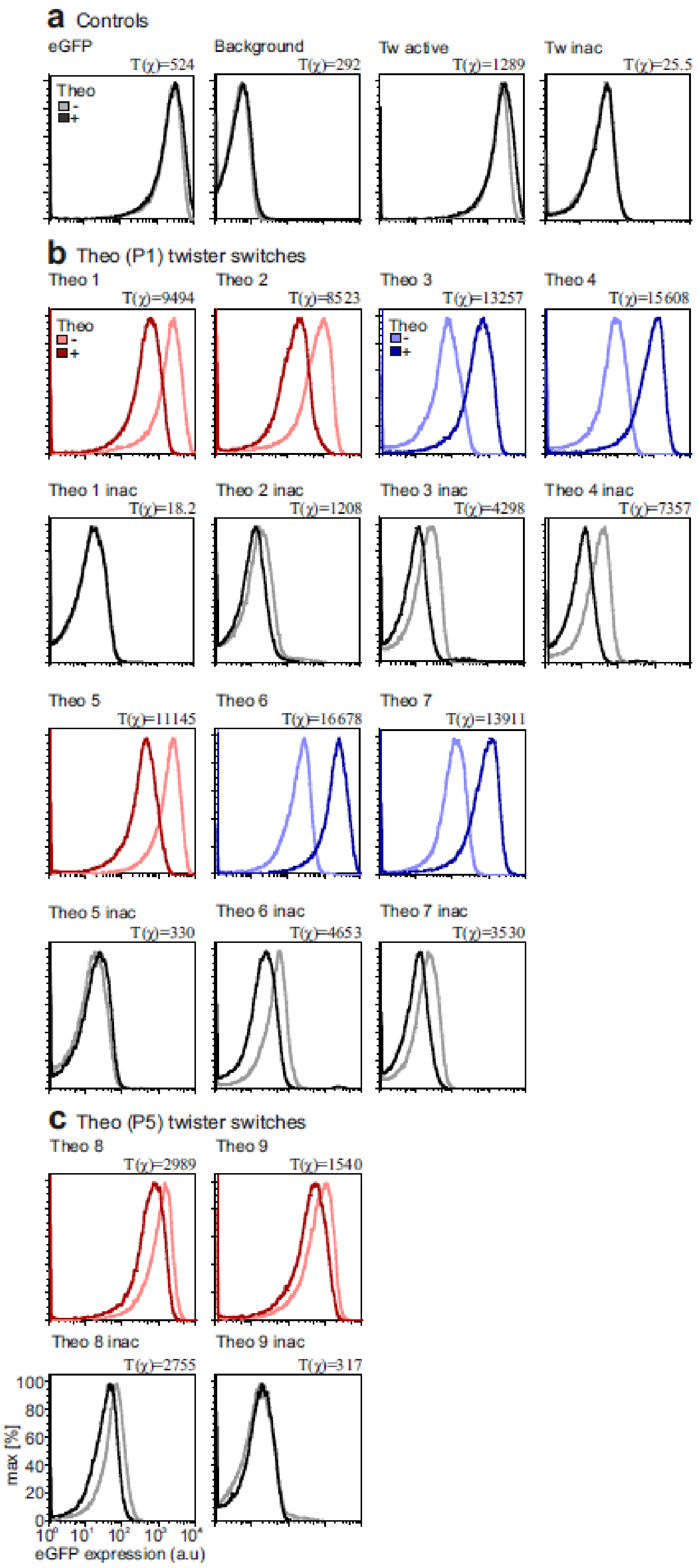

Supplementary Figure 7 | Flow cytometry (FC) analysis of the theophylline twister-based riboswitches. FC histograms of (**a**) the controls, (**b**) the theophylline (P1) switches and (**c**) the theophylline (P5) switches recorded in the presence (+) and in the absence (-) of 2.5 mM theophylline. The FC diagrams of the controls and of the catalytically inactive mutants are represented by grey tone traces, the diagrams of the off-switches by red tone traces and the ones of the on-switches by blue tone traces.

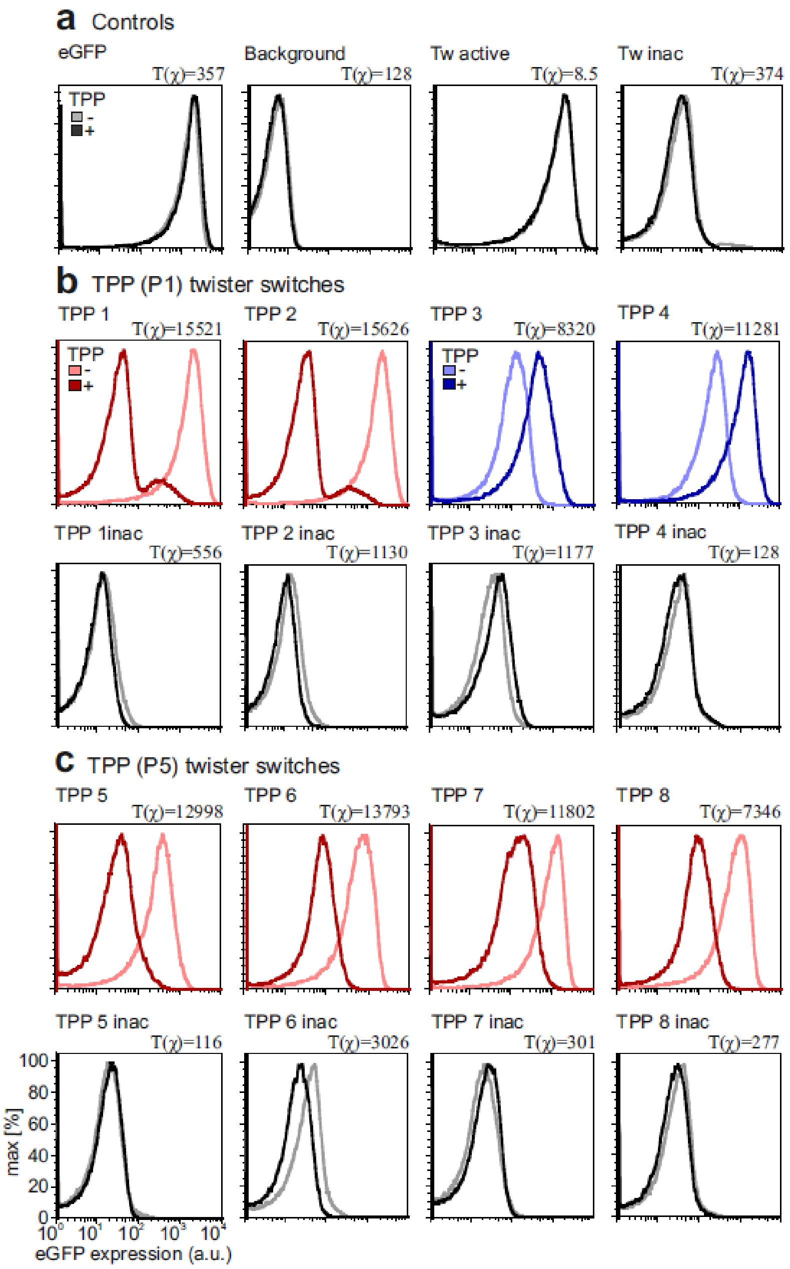

Supplementary Figure 8 | Flow cytometry (FC) analysis of the TPP twister-based riboswitches. FC histograms of (**a**) the controls, (**b**) the TPP (P1) switches and (**c**) the TPP (P5) switches recorded in the presence (+) and in the absence (-) of 1 mM thiamin. The FC diagrams of the controls and of the catalytically inactive mutants are represented by grey tone traces, the diagrams of the off-switches by red tone traces and the ones of the on-switches by blue tone traces.

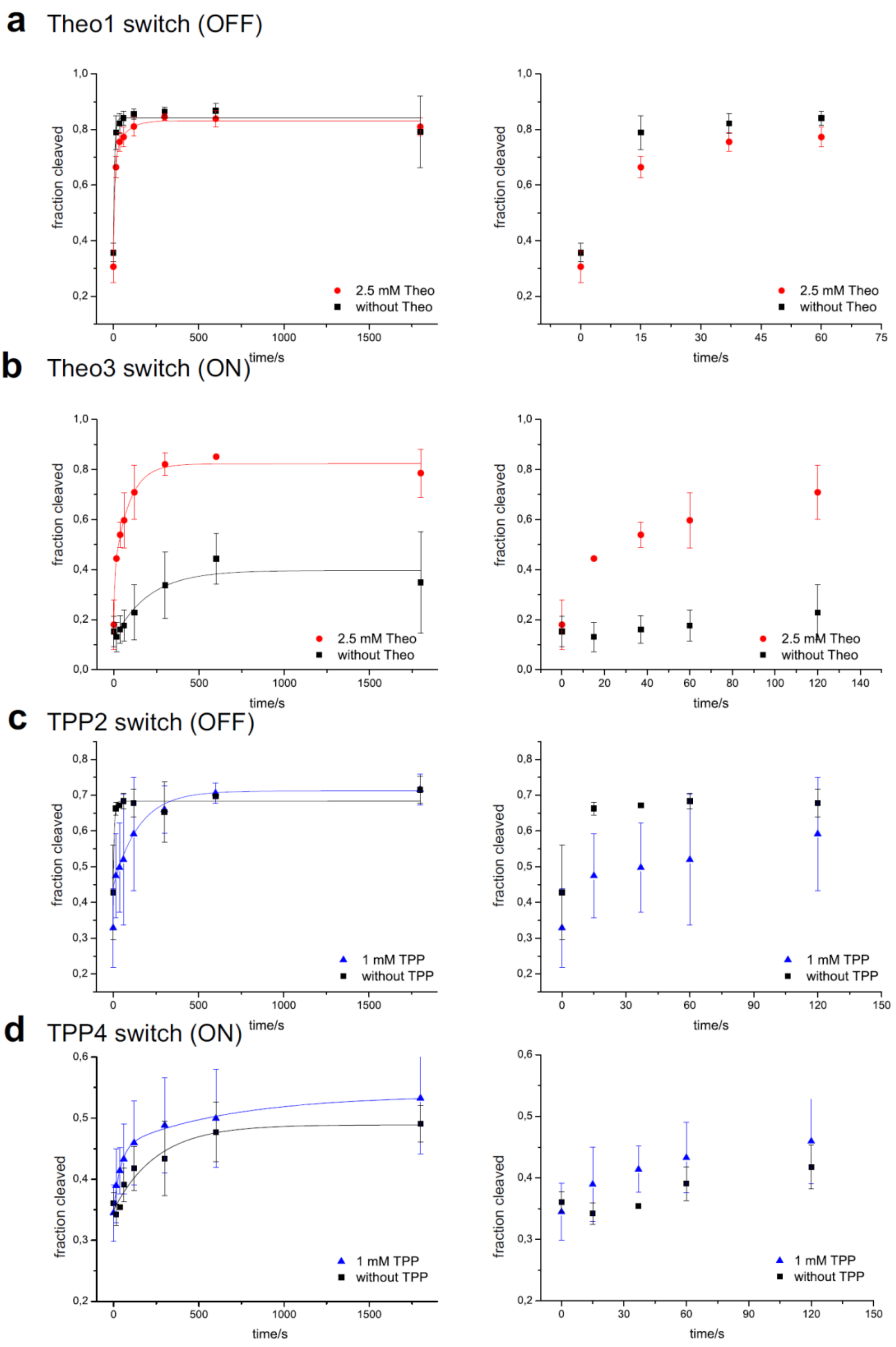

Supplementary Figure 9 | Kinetics of the four selected twister-based aptazymes. The kinetics were determined for (**a**) a theophylline-dependent off-switch (Theo1 - **Supplementary Fig. 4**), (**b**) a theophylline-dependent on-switch (Theo3 - **Supplementary Fig. 4**), (**c**) a TPP-dependent off-switch (TPP2 - **Supplementary Fig. 5**) and (**d**) a TPP-dependent on-switch (TPP4 - **Supplementary Fig. 5**) in 50 mM Tris-HCl pH 7.5, 1M KCl and 1 mM $MgCl_2$ in the presence and in the absence of the respective ligand (2.5 mM theophylline or 1 mM thiamin pyrophosphate – TPP). The final concentration of the RNA in the reaction mix was 50 nM. The kinetic traces were fitted with either mono- or bi-exponential functions. The error bars represent the standard deviation of independent triplicates. In the left column the full timecourse is shown. On the right, an enlarged region including only the first time points is represented.

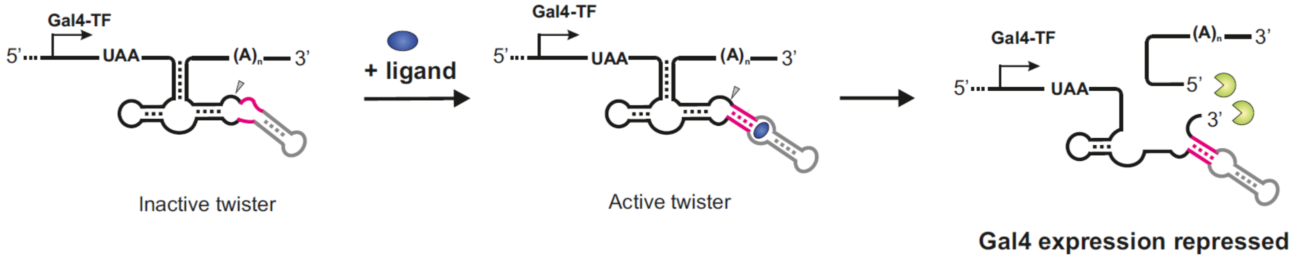

Supplementary Figure 10 | Mechanisms of twister-based off-switches in yeast. The twister-based aptazyme is inserted in the 3'-UTR of the reporter gene. The addition of the ligand to the growth medium increases the activity of the ligand-dependent twister promoting the removal of the poly(A) tail. This reduces the stability of the *GAL4* mRNA which is rapidly degraded in the cell and results in a lower reporter gene expression (off-switch).

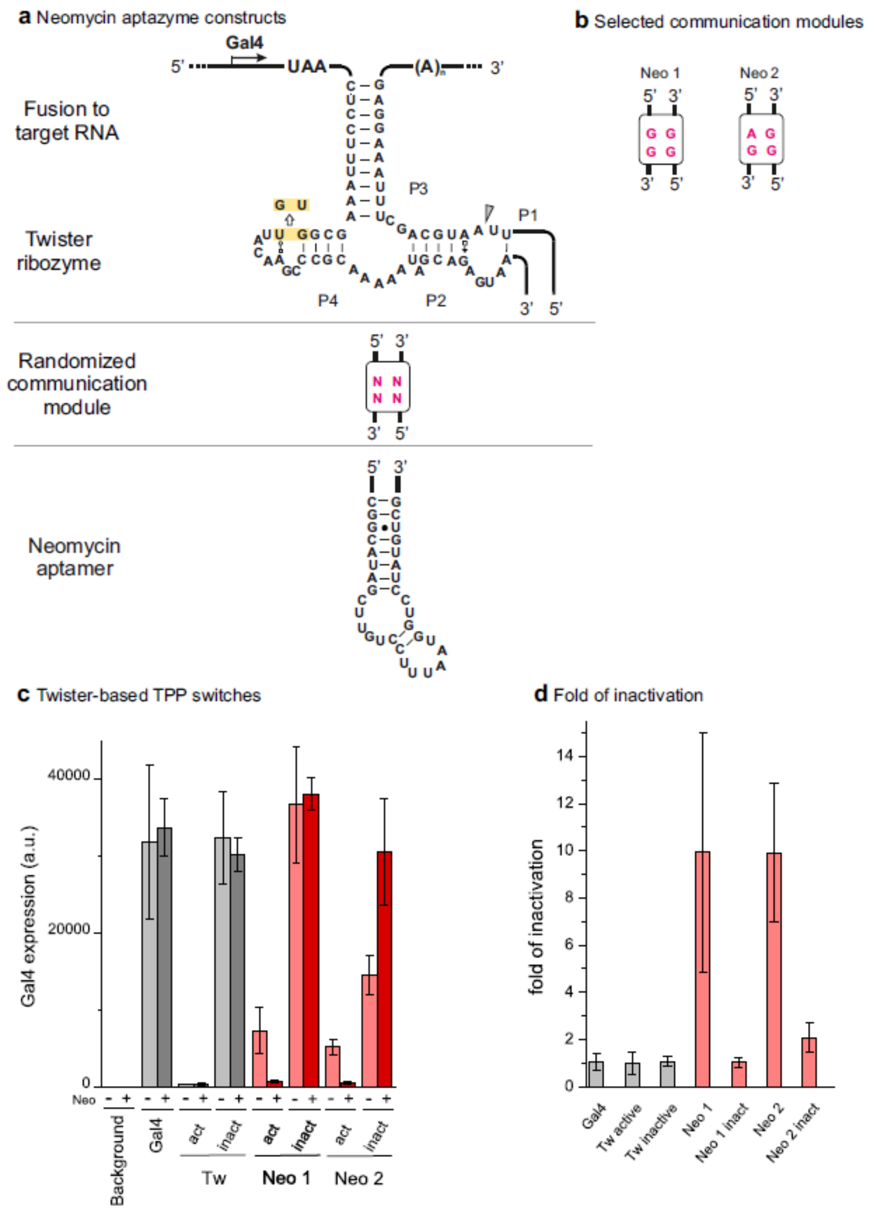

Supplementary Figure 11 | Design, sequences and performances of the neomycin twister-based riboswitches in yeast. (**a**) The neomycin P1 library was designed using a symmetric communication module of four randomized nucleotides. Cleavage-inactive control constructs were generated by inverting two conserved nucleotides highlighted in yellow. The cleavage site is indicated with a grey arrow. (**b**) The identified communication modules of the screened neomycin riboswitches. (**c**) Levels of β-galactosidase expression of the selected neomycin clones in the absence (-) and in the presence (+) of 100 µg/mL neomycin. The levels of reporter gene expression in the different conditions are represented by gray tone bars for the controls and red tone bars for the off-switches. The error bars represent standard deviations calculated on independent biological triplicates (**d**) The performances (fold of inactivation) of the off-switches were calculated as the ratio of chemoluminescence of active divided by inactive expression state and are represented with red bars. The error bars represent standard deviations calculated on independent biological triplicates

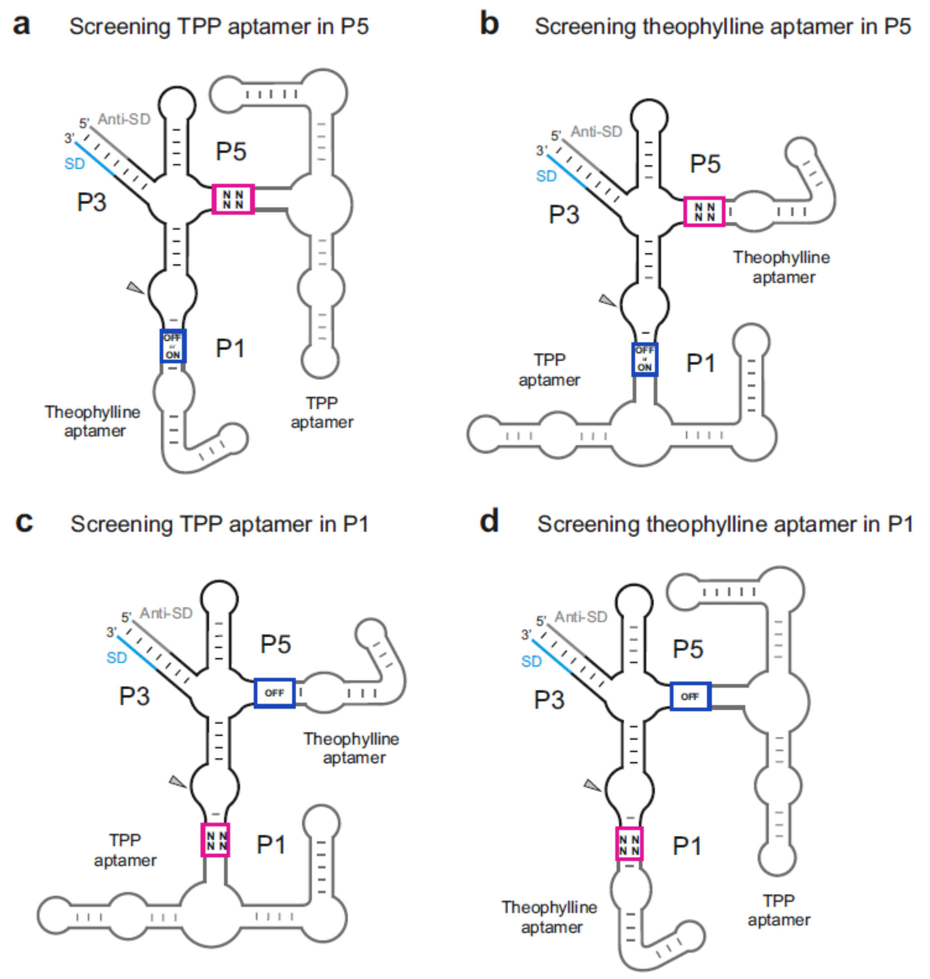

Supplementary Figure 12 | Screening approach for the development of two-input artificial riboswitches. We started from the P1 or P5 theophylline and TPP riboswitches and performed a screening of libraries generated by randomizing the communication module in the other position. We screened a total of six types of libraries: (**a**) P1-theophylline-off/P5-TPP randomized and P1-theophylline-on/P5-TPP randomized (**b**) P1-TPP-off/P5-theophylline randomized and P1-TPP-on/P5-theophylline randomized (**c**) P5-theophylline-off/P1-TPP randomized (**d**) P5-TPP-off/P1-theophylline randomized.

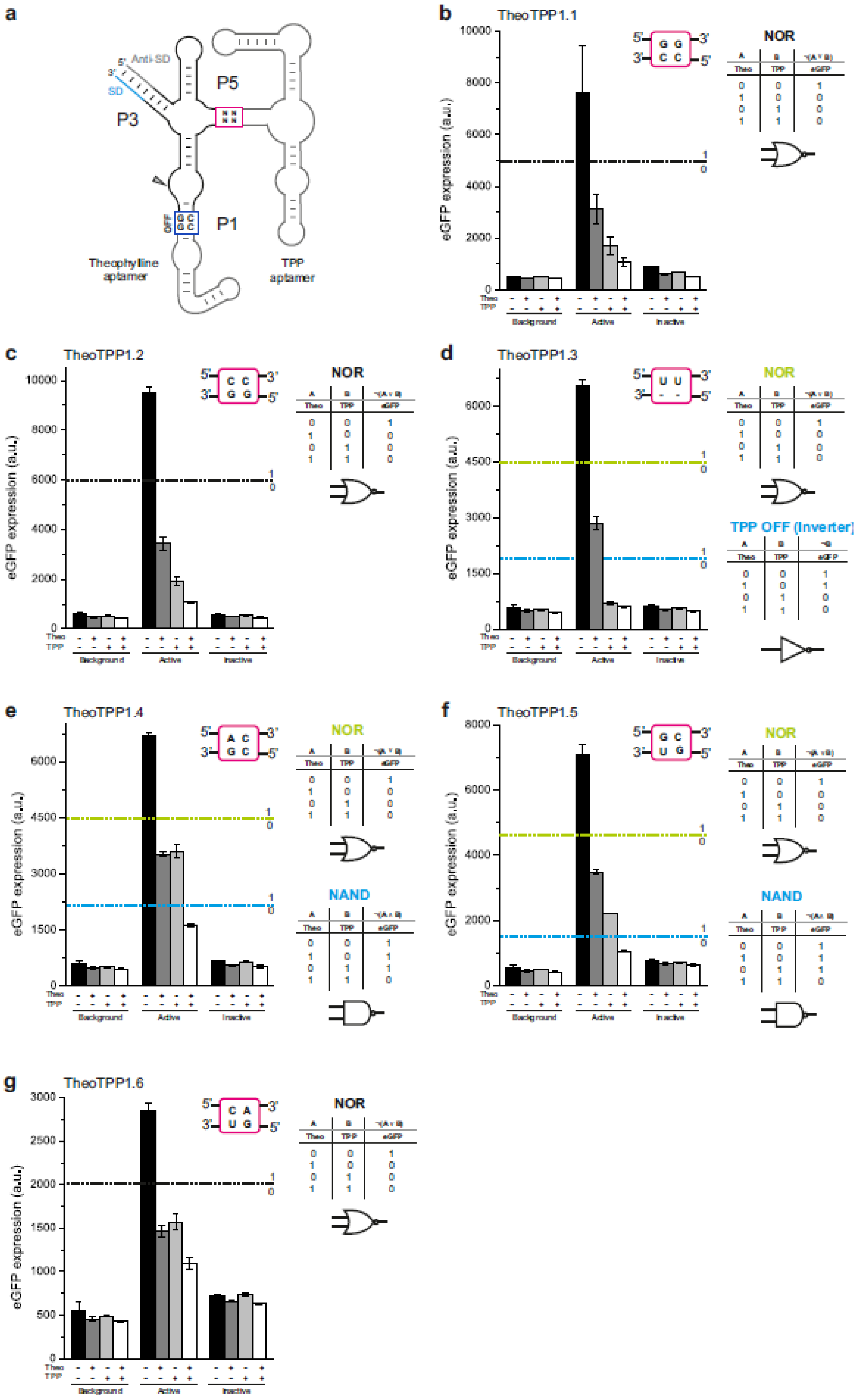

Supplementary Figure 13 | Results for screening library 1. (**a**) Schematic representation of the twister-based two-input riboswitches generated by screening of the library 1. In blue the communication module of the Theo1 off-switch. The randomized communication module in P5 is represented in magenta. (**b-g**) The P5 communication modules, eGFP expression levels, table of truth and logic gate symbols of the selected Boolean logic gates are shown. The background fluorescence and the eGFP expression levels of the catalytically inactive mutants are also reported. The threshold values, which are represented with dashed lines, allow defining high (value 1) and low (value 0) eGFP expression. Depending on where the threshold is set, some switches can be assigned to one or to another category. In these cases the two alternative threshold values are represented in green and blue respectively.

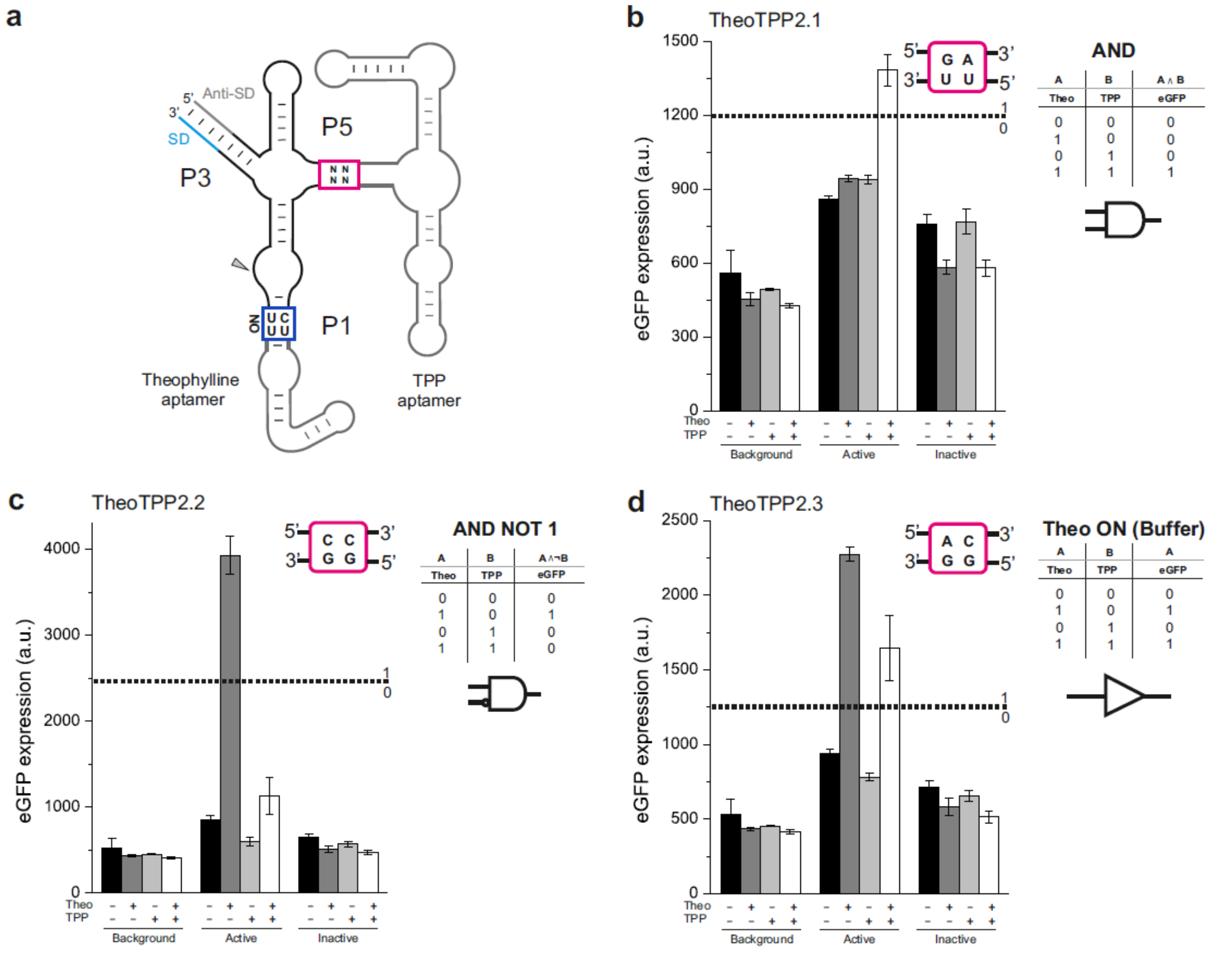

Supplementary Figure 14 | Results for screening library 2. (**a**) Schematic representation of the twister-based two-input riboswitches generated by screening of the library 2. In blue the communication module of the Theo3 on switch. The randomized communication module in P5 is represented in magenta. (**b-d**) The P5 communication modules, eGFP expression levels, table of truth and logic gate symbols of the selected Boolean logic gates are shown. The background fluorescence and the eGFP expression levels of the catalytically inactive mutants are also reported. The threshold values, which are represented with dashed lines, allow defining high (value 1) and low (value 0) eGFP expression.

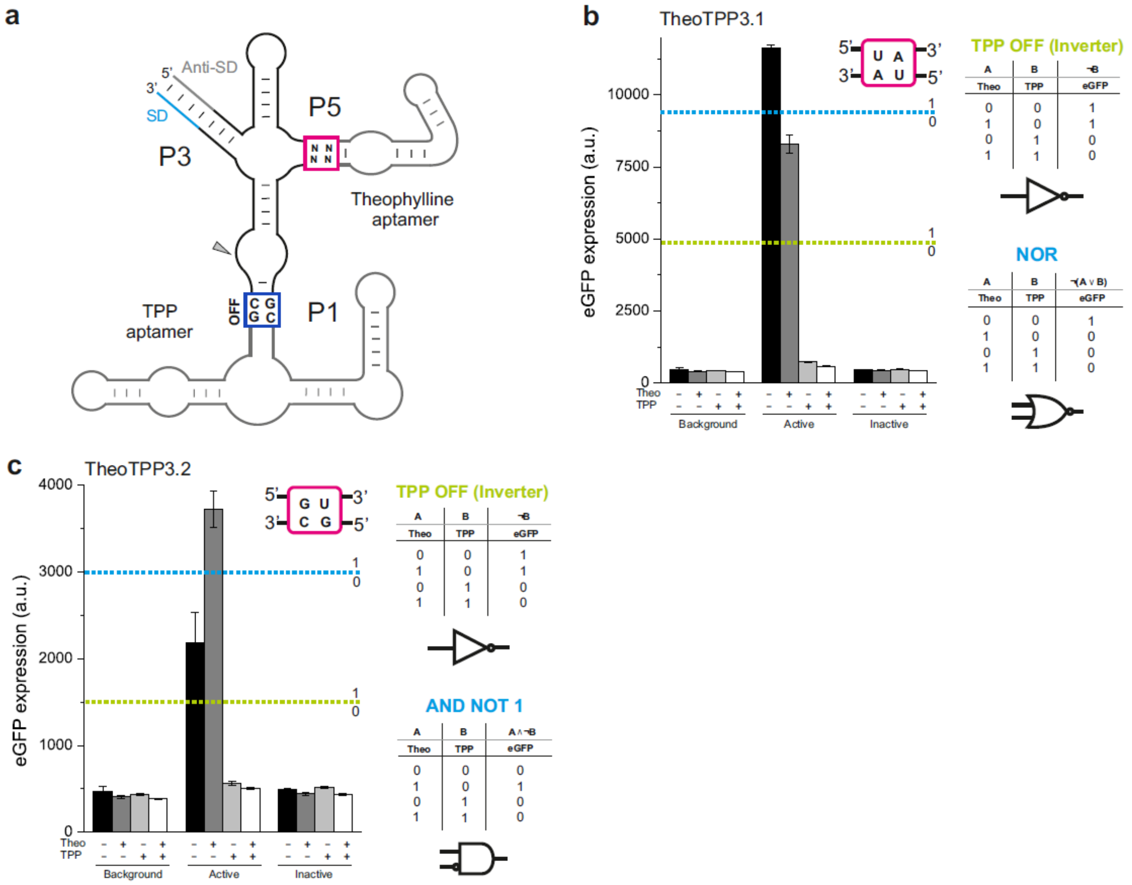

Supplementary Figure 15 | Results for screening library 3. (**a**) Schematic representation of the twister-based two-input riboswitches generated by screening of the library 3. In blue the communication module of the TPP2 off-switch. The randomized communication module in P5 is represented in magenta. (**b,c**) The P5 communication modules, eGFP expression levels, table of truth and logic gate symbols of the selected Boolean logic gates are shown. The background fluorescence and the eGFP expression levels of the catalytically inactive mutants are also reported. The threshold values, which are represented with dashed lines, allow defining high (value 1) and low (value 0) eGFP expression. Depending on where the threshold is set, the switches can be assigned to one or to another category. The two alternative threshold values are represented in green and blue respectively.

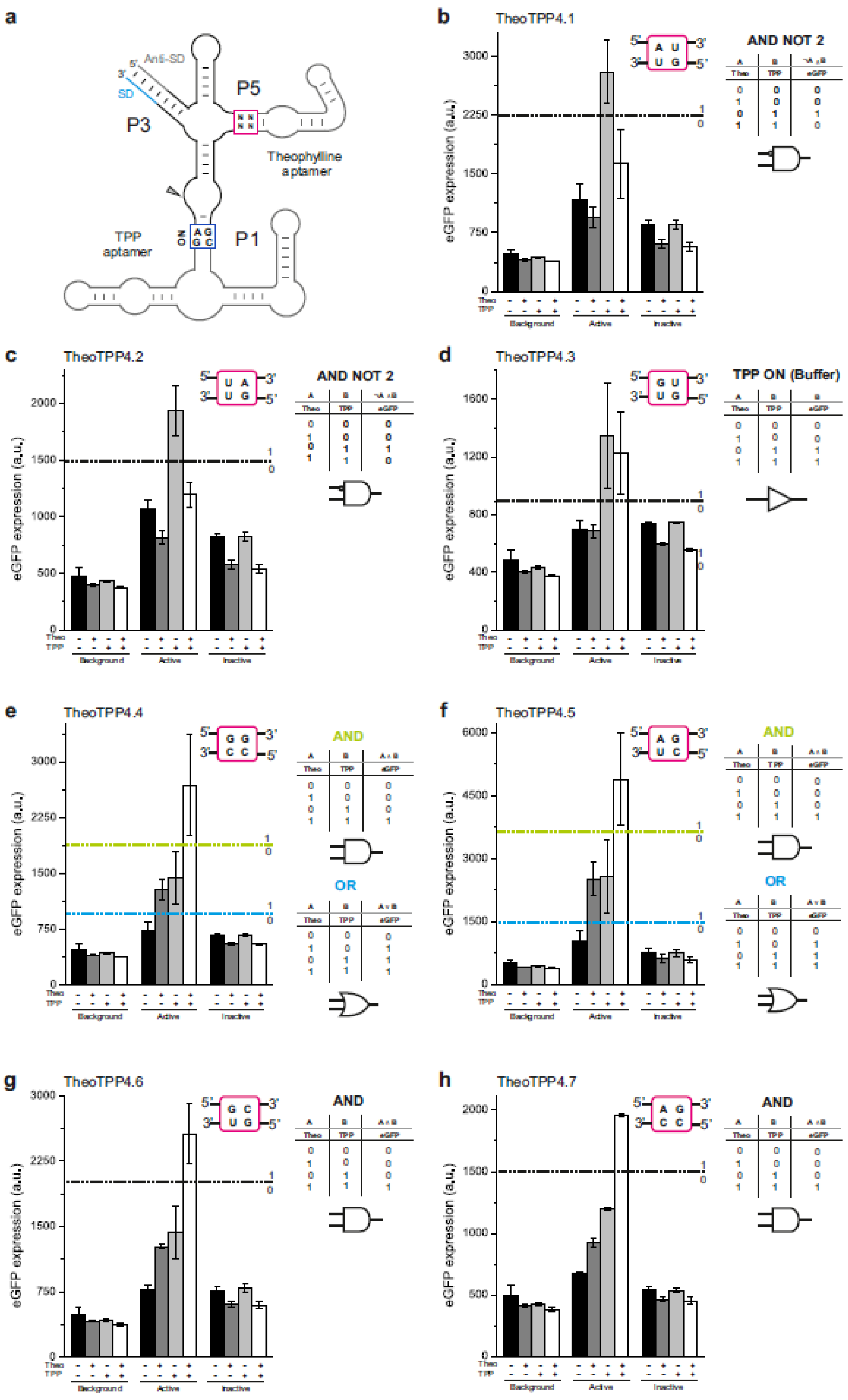

Supplementary Figure 16 | Results for screening library 4. (**a**) Schematic representation of the twister-based two-input riboswitches generated by screening of the library 4. In blue the communication module of the TPP4 on-switch. The randomized communication module in P5 is represented in magenta. (**b-h**) The P5 communication modules, eGFP expression levels, table of truth and logic gate symbols of the selected Boolean logic gates are shown. The background fluorescence and the eGFP expression levels of the catalytically inactive mutants are also reported. The threshold values, which are represented with dashed lines, allow defining high (value 1) and low (value 0) eGFP expression. Depending on where the threshold is set, some switches can be assigned to one or to another category. In these cases the two alternative threshold values are represented in green and blue respectively.

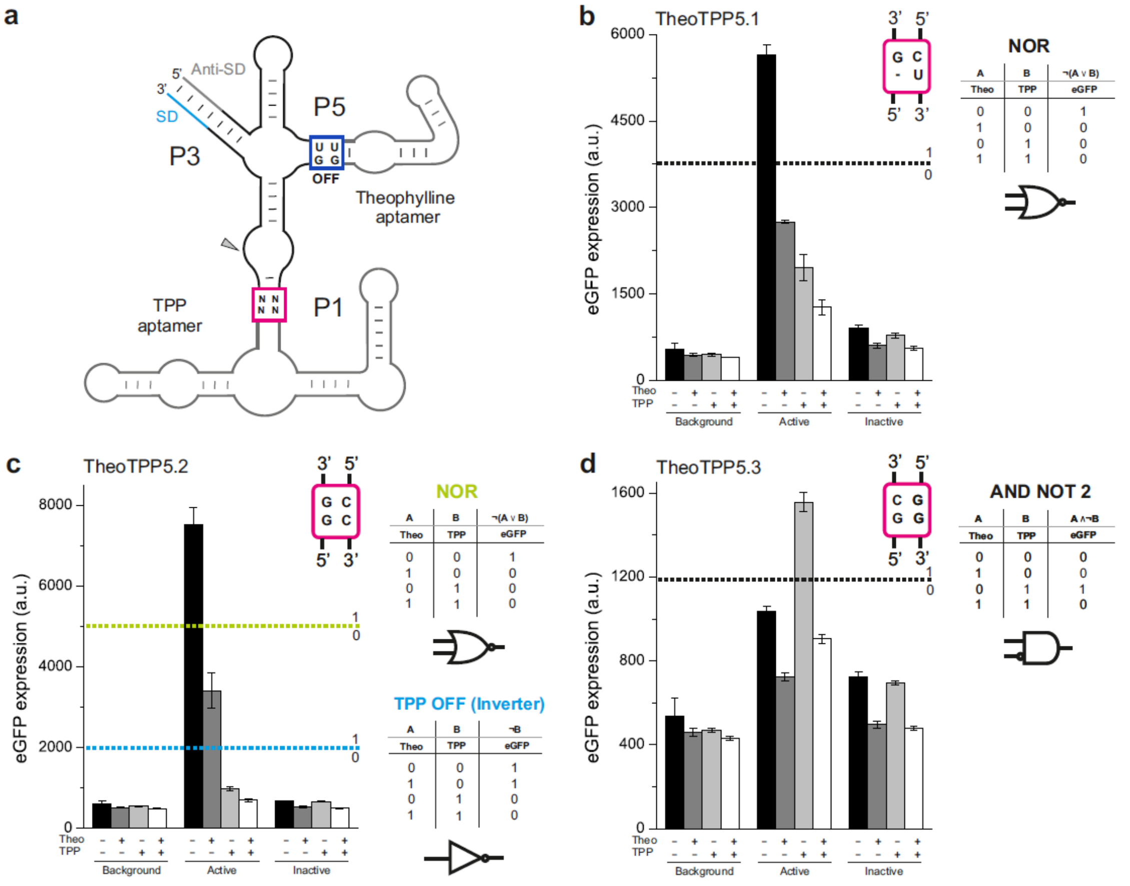

Supplementary Figure 17| Results for screening library 5. (**a**) Schematic representation of the twister-based two-input riboswitches generated by screening of the library 5. In blue the communication module of the Theo9 off-switch. The randomized communication module in P1 is represented in magenta. (**b-d**) The P1 communication modules, eGFP expression levels, table of truth and logic gate symbols of the selected Boolean logic gates are shown. The background fluorescence and the eGFP expression levels of the catalytically inactive mutants are also reported. The threshold values, which are represented with dashed lines, allow defining high (value 1) and low (value 0) eGFP expression. Depending on where the threshold is set, some switches can be assigned to one or to another category. In these cases the two alternative threshold values are represented in green and blue respectively.

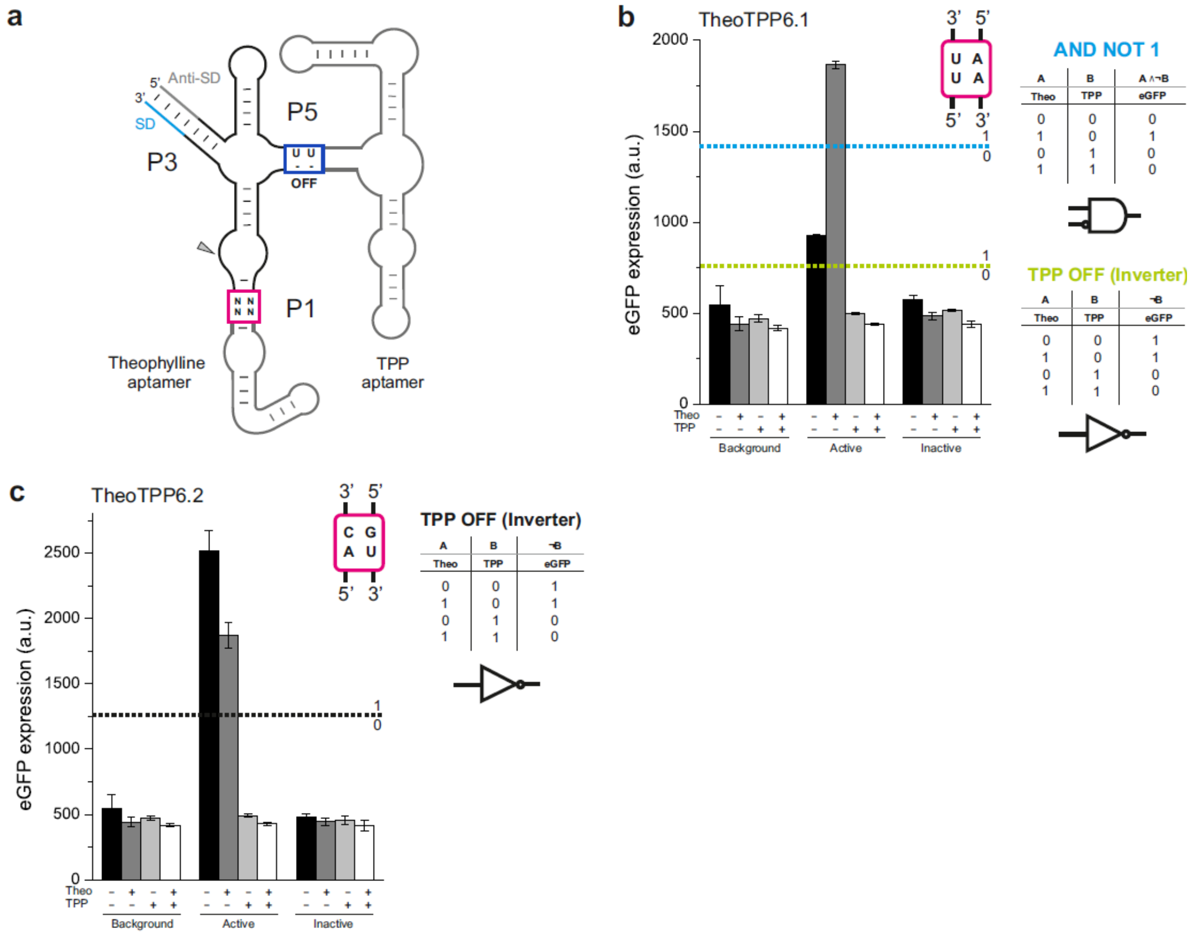

Supplementary Figure 18 | Results for screening library 6. (**a**) Schematic representation of the twister-based two-input riboswitches generated by screening of the library 6. In blue the communication module of the TPP5 off-switch. The randomized communication module in P1 is represented in magenta. (**b**,**c**) The P1 communication modules, eGFP expression levels, table of truth and logic gate symbols of the selected Boolean logic gates are shown. The background fluorescence and the eGFP expression levels of the catalytically inactive mutants are also reported. The threshold values, which are represented with dashed lines, allow defining high (value 1) and low (value 0) eGFP expression. Depending on where the threshold is set, some switches can be assigned to one or to another category. In these cases the two alternative threshold values are represented in green and blue respectively.

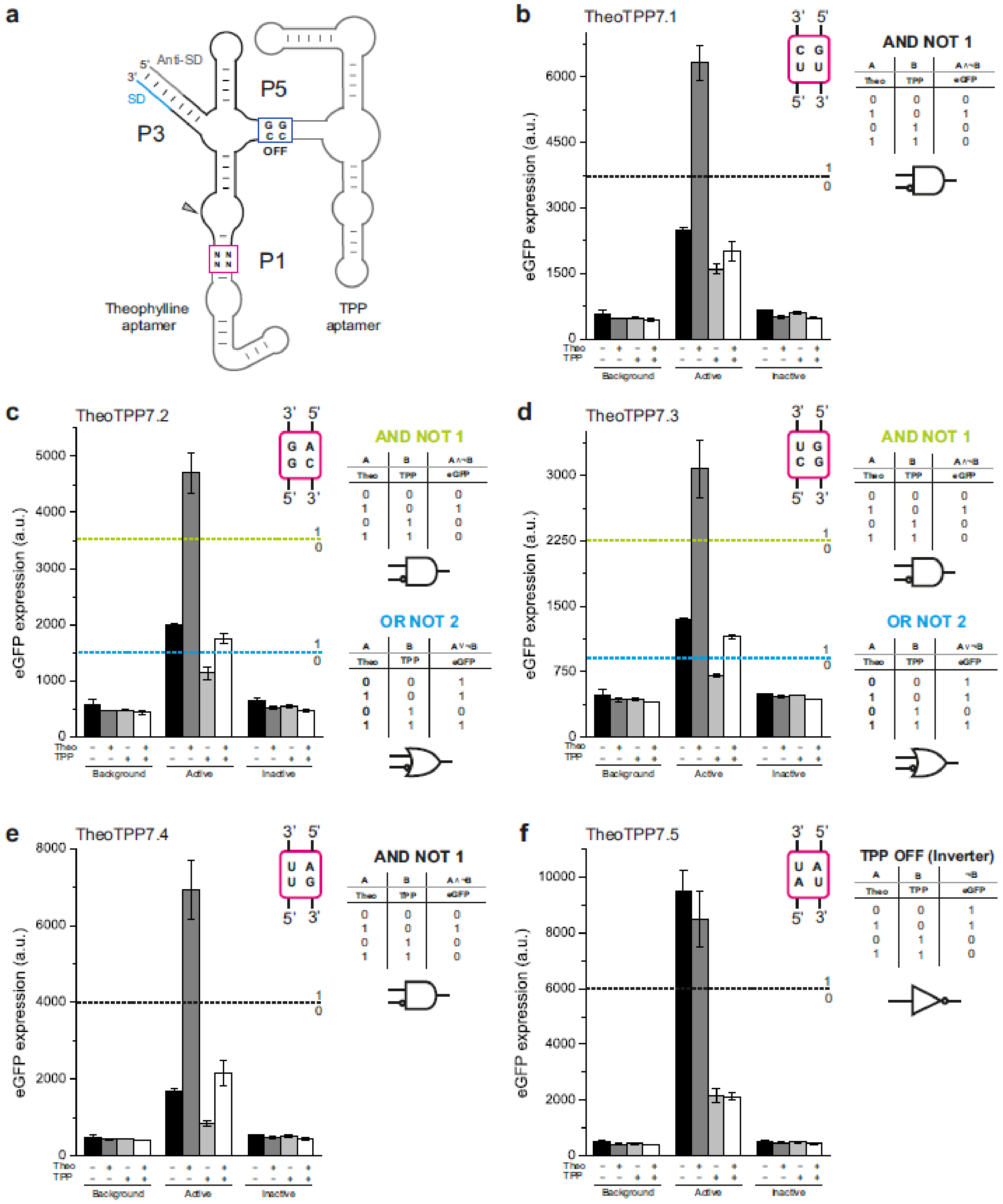

Supplementary Figure 19 | Results for screening library 7. (**a**) Schematic representation of the twister-based two-input riboswitches generated by screening of the library 6. In blue the communication module of the TPP7 off-switch. The randomized communication module in P1 is represented in magenta. (**b-f**) The P1 communication modules, eGFP expression levels, table of truth and logic gate symbols of the selected Boolean logic gates are shown. The background fluorescence and the eGFP expression levels of the catalytically inactive mutants are also reported. The threshold values, which are represented with dashed lines, allow defining high (value 1) and low (value 0) eGFP expression. Depending on where the threshold is set, some switches can be assigned to one or to another category. In these cases the two alternative threshold values are represented in green and blue respectively.

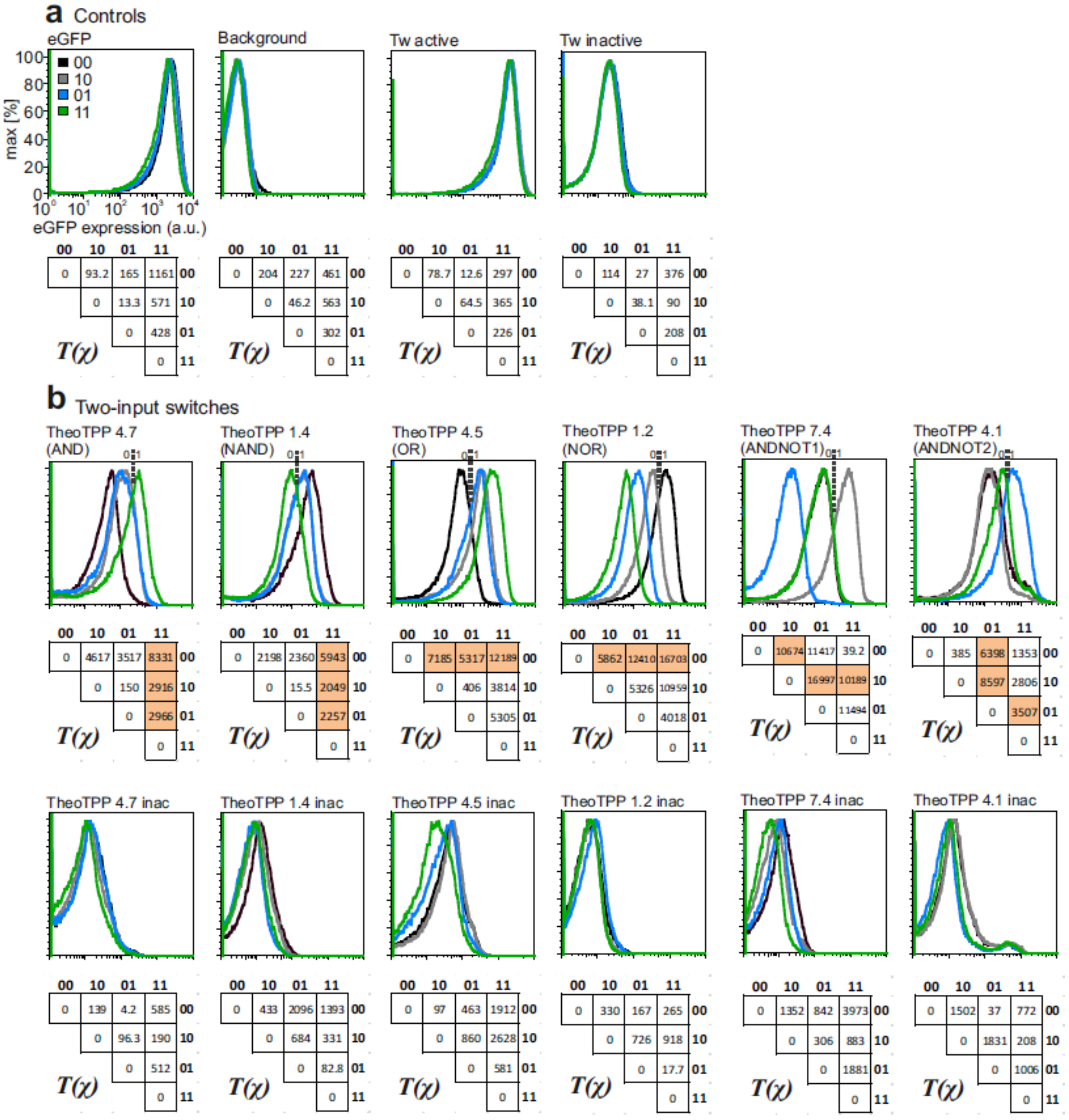

Supplementary Figure 20 | Flow cytometry (FC) analysis of selected two-input twister-based riboswitches. FC histograms of (**a**) the controls and (**b**) the two-input switches recorded in the absence of ligand (black traces - 0 0), in the presence of 2.5 mM theophylline (grey traces - 1 0), 1 mM thiamin (blue traces - 0 1) and 2.5 mM theophylline/1 mM thiamin (green traces - 1 1). The threshold fluorescence values that define the logic gates are shown with a dashed line. T(X) values were calculated for each couple of histograms and are shown below the FC panels. The relevant T(X) values for the definition of the logic gate are highlighted in orange.

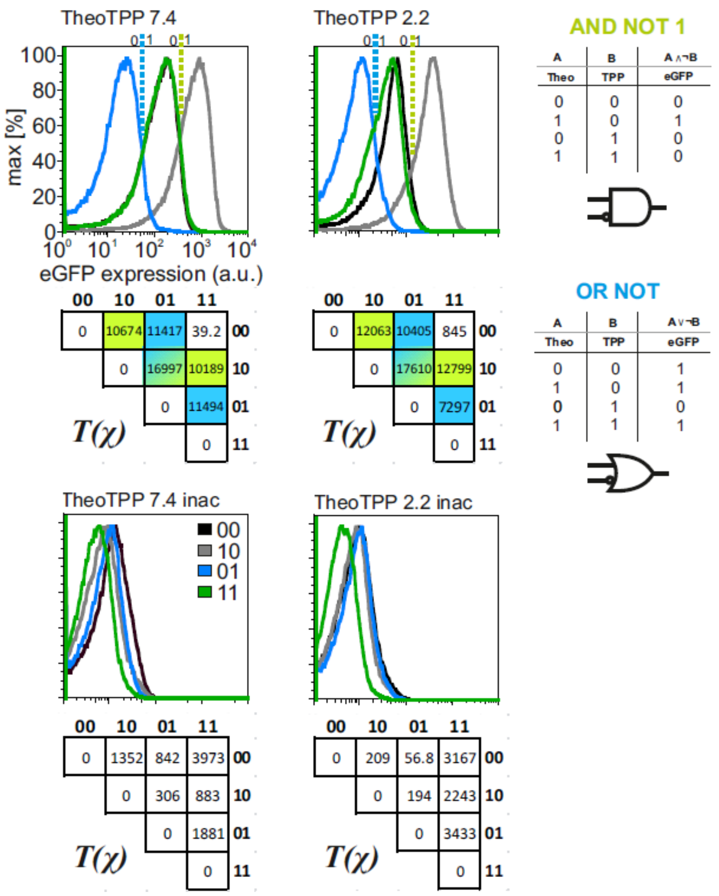

Supplementary Figure 21 | Flow cytometry (FC) analysis of the TheoTPP7.4 and of the TheoTPP2.2 two-input riboswitches. FC histograms of the two-input switches recorded in the absence of ligand (black traces - 0 0), in the presence of 2.5 mM theophylline (grey traces - 1 0), 1 mM thiamin (blue traces - 0 1) and 2.5 mM theophylline/1 mM thiamin (green traces - 1 1). The threshold fluorescence values that define the two alternative logic gates (ANDNOT1 and ORNOT) are shown with green and blue dashed lines. T(X) values were calculated for each couple of histograms and are shown below the FC panels. The relevant T(X) values for the definition of the logic gate are highlighted in green and blue.

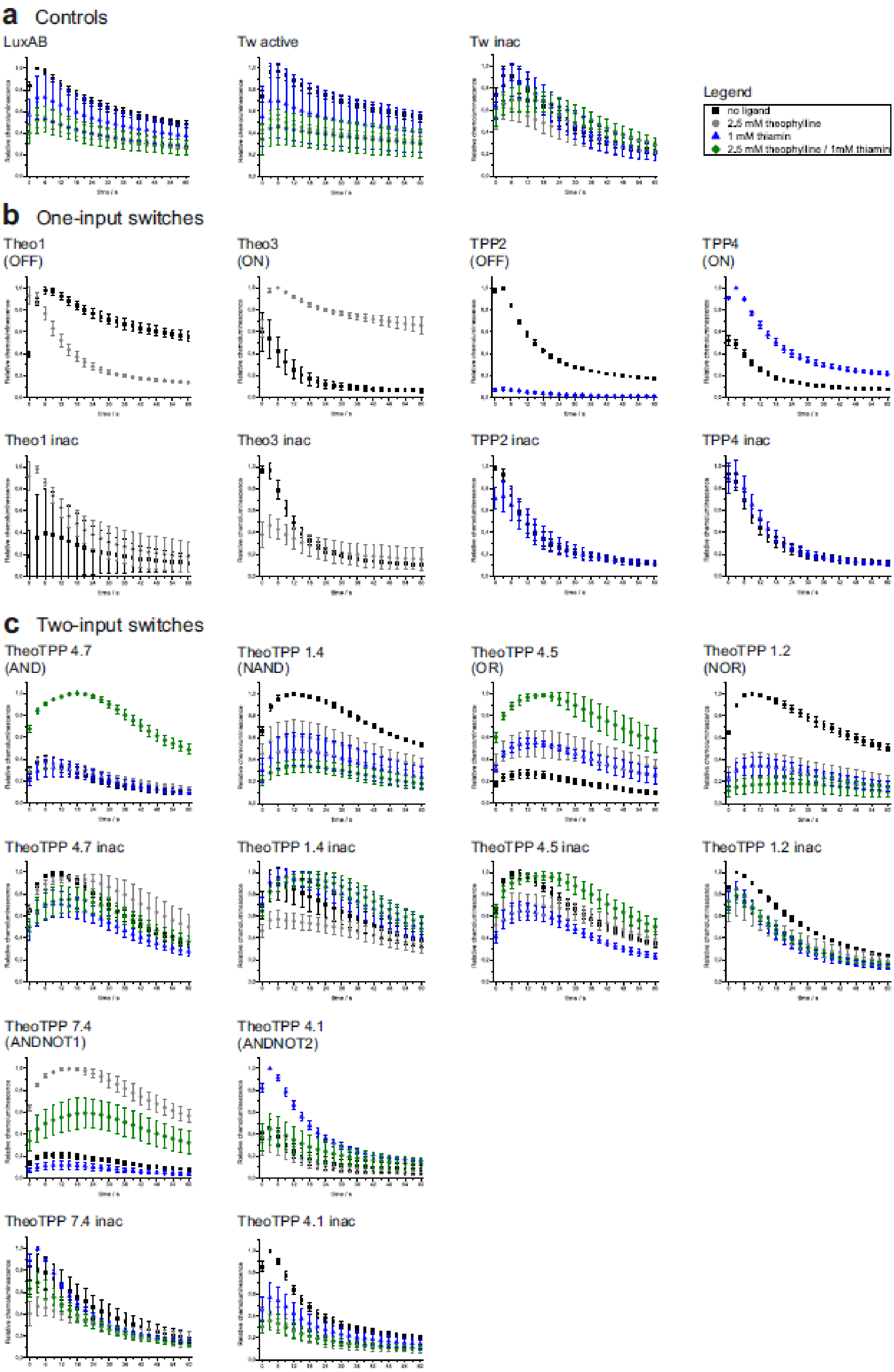

Supplementary Figure 22 | Bioluminescence analysis of the one- and two-input riboswitches using bacterial luciferase (luxAB) as a reporter gene. Chemoluminescence was acquired every three seconds for a total of one minute after the addition of 10 µL of 0.01% decanal in ethanol solution to 100 µL of bacterial culture and using an integration time of 2000 ms. In (**a**) the chemoluminescence traces of the positive control (LuxAB) and of the twister active and inactive type P3 SD constructs. In (**b**) the data of the theophylline and thiamin switches and the catalytically inactive controls. In (**c**) the traces of the two-input riboswitches and of the relative catalytically inactive controls. The chemoluminescence traces were recorded in the absence of ligand (black squares), in the presence of 2.5 mM theophylline (grey circles), 1 mM thiamin (blue triangle) and 2.5 mM theophylline/1 mM thiamin (green diamonds).

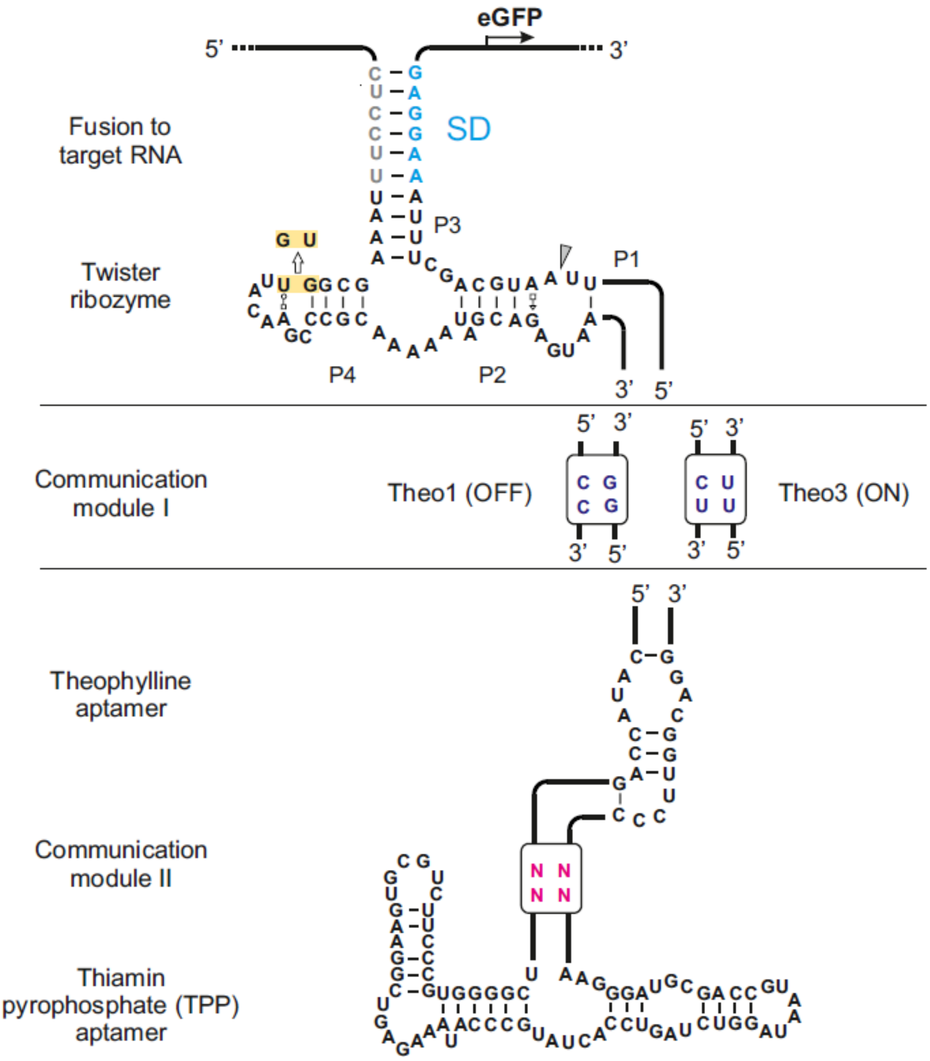

Supplementary Figure 23 | Screening approach for the development of artificial riboswitches carrying two aptamer connected in series. We performed a screening of two libraries generated starting from Theo1 (OFF) and Theo3 (ON) switches and connecting the TPP aptamer to the terminal stem-loop of the theophylline aptamer through a four nucleotide randomized communication module.

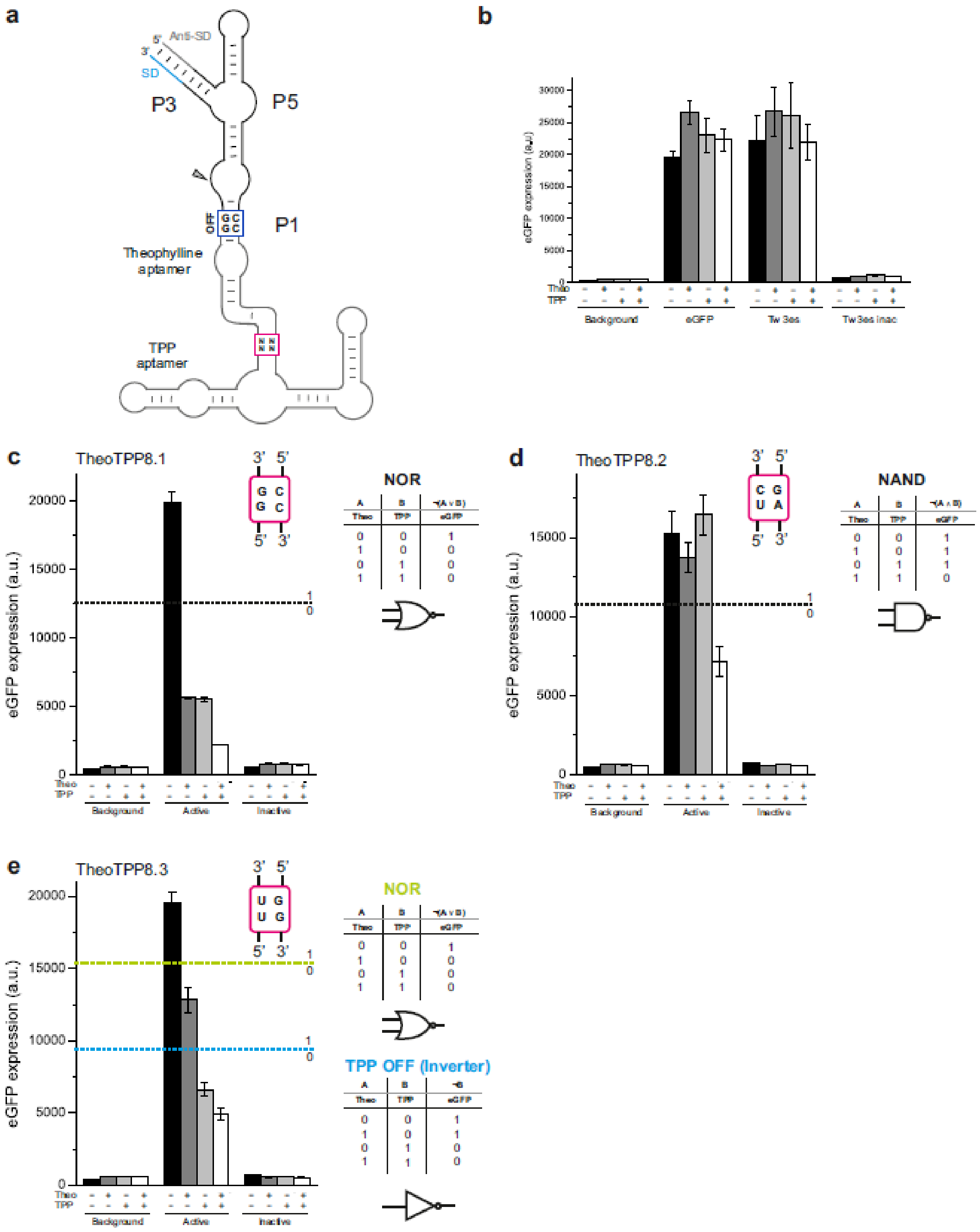

Supplementary Figure 24 | Results for screening library 8. (**a**) Schematic representation of the twister-based two-input riboswitches generated by screening of the library 8. In blue the communication module of the Theo1 off-switch. The randomized sequence connecting the TPP aptamer to the theophylline aptamer is represented in magenta. (**b-e**) The TPP aptamer communication modules, eGFP expression levels, table of truth and logic gate symbols of the selected Boolean logic gates are shown. The background fluorescence and the eGFP expression levels of the catalytically inactive mutants are also reported. The threshold values, which are represented with dashed lines, allow defining high (value 1) and low (value 0) eGFP expression. Depending on where the threshold is set, some switches can be assigned to one or to another category. In these cases the two alternative threshold values are represented in green and blue respectively.

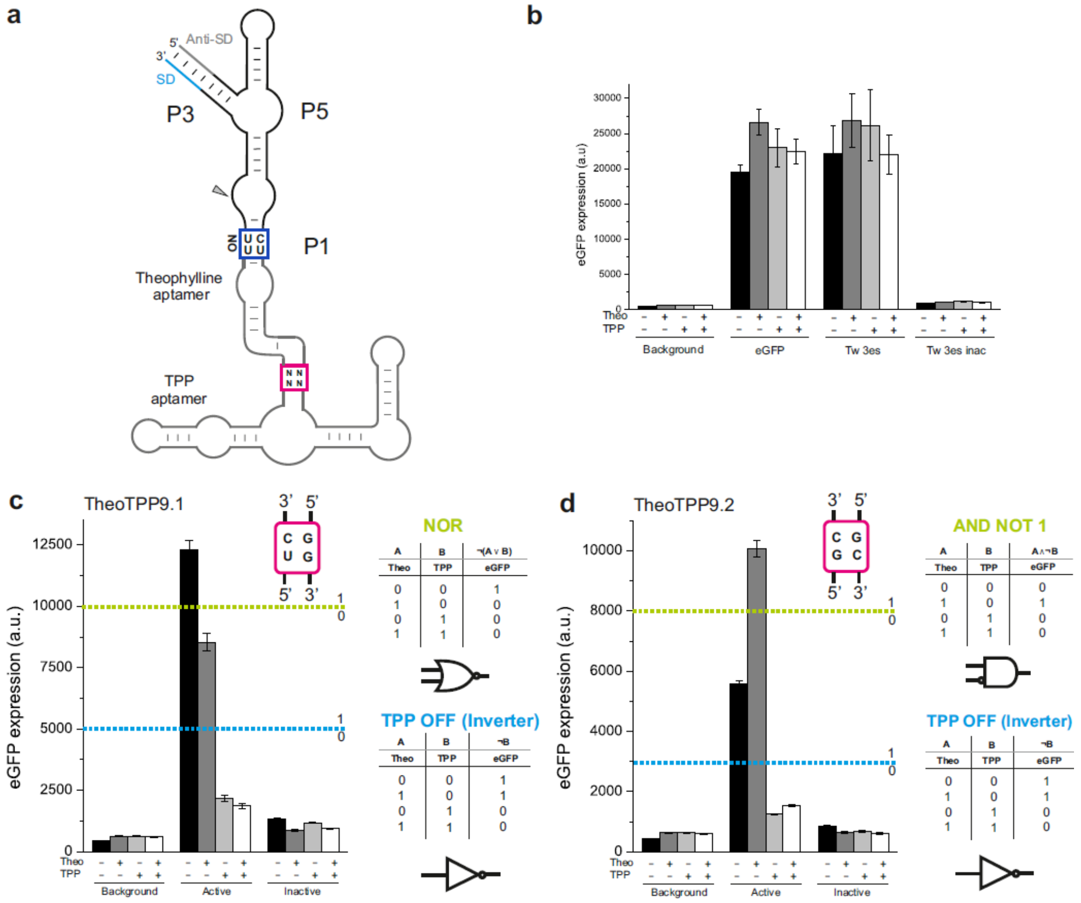

Supplementary Figure 25 | Results for screening library 9. (**a**) Schematic representation of the twister-based two-input riboswitches generated by screening of the library 9. In blue the communication module of the Theo3 on-switch. The randomized sequence connecting the TPP aptamer to the theophylline aptamer is represented in magenta. (**b-d**) The TPP aptamer communication modules, eGFP expression levels, table of truth and logic gate symbols of the selected Boolean logic gates are shown. The background fluorescence and the eGFP expression levels of the catalytically inactive mutants are also reported. The threshold values, which are represented with dashed lines, allow defining high (value 1) and low (value 0) eGFP expression. Depending on where the threshold is set, some switches can be assigned to one or to another category. In these cases the two alternative threshold values are represented in green and blue respectively.

Controls			
**eGFP**	84,1	**eGFP**	80,9
**eGFP_Theo**	87,7	**eGFP_TPP**	78,7
**Background**	80,6	**Background**	80,8
**Background_Theo**	86,1	**Background_TPP**	78,7
**Twister active**	81,2	**Twister active**	78,3
**Twister active_Theo**	86,8	**Twister active_TPP**	79,3
**Twister inactive**	80,4	**Twister inactive**	78,9
**Twister inactive_Theo**	86,5	**Twister inactive_TPP**	78,8
**Twister-based riboswitches**			
**Theo 1**	75,9	**TPP 1**	78,2
**Theo 1_Theo**	76,9	**TPP 1_TPP**	81,8
**Theo 1i**	73,2	**TPP 1i**	78,1
**Theo 1i_Theo**	73,4	**TPP 1i_TPP**	77,9
**Theo 2**	80,9	**TPP 2**	75,2
**Theo 2_Theo**	85,3	**TPP 2_TPP**	80,6
**Theo 2i**	79,7	**TPP 2i**	79,3
**Theo 2i_Theo**	84,8	**TPP 2i_TPP**	79,4
**Theo 3**	79,4	**TPP 3**	79,6
**Theo 3_Theo**	82,6	**TPP 3_TPP**	77,7
**Theo 3i**	80	**TPP 3i**	79,7
**Theo 3i_Theo**	85,9	**TPP 3i_TPP**	80,2
**Theo 4**	80,1	**TPP 4**	78,2
**Theo 4_Theo**	86,4	**TPP 4_TPP**	79,7
**Theo 4i**	79,9	**TPP 4i**	81,6
**Theo 4i_Theo**	85,3	**TPP 4i_TPP**	79,4
**Theo 5**	76,4	**TPP 5**	81,7
**Theo 5_Theo**	72,8	**TPP 5_TPP**	81,3
**Theo 5i**	70,3	**TPP 5i**	77,6
**Theo 5i_Theo**	73,5	**TPP 5i_TPP**	82,6
**Theo 6**	80,4	**TPP 6**	79,9
**Theo 6_Theo**	85,8	**TPP 6_TPP**	80
**Theo 6i**	78,5	**TPP 6i**	81,8
**Theo 6i_Theo**	84,8	**TPP 6i_TPP**	79,6
**Theo 7**	78,5	**TPP 7**	81,2
**Theo 7_Theo**	84,9	**TPP 7_TPP**	79,3
**Theo 7i**	77,2	**TPP 7i**	75,7
**Theo 7i_Theo**	84,3	**TPP 7i_TPP**	82,8
**Theo 8**	79,9	**TPP 8**	82
**Theo 8_Theo**	84,3	**TPP 8_TPP**	80,1
**Theo 8i**	80,6	**TPP 8i**	78,9
**Theo 8i_Theo**	85,5	**TPP 8i_TPP**	79,4
**Theo 9**	79,6		
**Theo 9_Theo**	74,2		
**Theo 9i**	75,4		
**Theo 9i_Theo**	79,4		

Supplementary Table 1 | Percentages of events included in the gated flow cytometry populations of the one-input twister-based riboswitches. The values of the controls are also included.

		y_0	A	t (s)	k (min^{-1})	R^2
**Theo1**	*w/o ligand*	0.84	-0.48	6.81	8.81	0.971
	w/ ligand	0.81	-0.50	13.62	4.41	0.976
**Theo3**	*w/o ligand*	0.40	-0.28	201.47	0.30	0.884
	w/ ligand	0.81	-0.58	51.45	1.17	0.951
**TPP2**	*w/o ligand*	0.68	-0.26	6.04	9.94	0.946
	w/ ligand	0.70	-0.32	94.02	0.64	0.926
**TPP4**	*w/o ligand*	0.49	-0.14	254.34	0.24	0.929
	w/ ligand	0.51	-0.15	94.89	0.63	0.936

Supplementary Table 2 | Fitting parameters of the kinetics traces using a mono-exponential function. The selected aptazyme were tested in the absence and in the presence of the respective ligand.

		y_0	A_1	t_1 (s)	k_1 (min^{-1})	A_2	t_2 (s)	k_2 (min^{-1})	R^2
**Theo1**	*w/o ligand*	0.84	-0.24	11.18	5.37	-0.24	0.59	102.18	0.953
	w/ ligand	0.83	-0.14	63.05	0.95	-0.39	7.94	7.56	0.992
**Theo3**	*w/o ligand*	0.40	-0.14	201.47	0.30	-0.14	201.46	0.30	0.807
	w/ ligand	0.82	-0.20	4.65	12.89	-0.44	87.20	0.69	0.984
**TPP2**	*w/o ligand*	0.68	-0.13	6.04	9.94	-0.13	6.04	9.94	0.910
	w/ ligand	0.71	-0.12	0.34	177.15	-0.27	150.23	0.40	0.991
**TPP4**	*w/o ligand*	0.49	-0.11	368.99	0.16	-0.04	92.42	0.65	0.889
	w/ ligand	0.54	-0.10	39.63	1.51	-0.09	655.43	0.09	0.990

Supplementary Table 3 | Fitting parameters of the kinetics traces using a bi-exponential function. The selected aptazyme were tested in the absence and in the presence of the respective ligand.

Theo 1	CUCCUUUAAAGCGGUUACAAGCCCGCAAAAAUAGCAGAGUAACCCAUACCAGCCGAAAGGCCCUUGGCAGGGGUUAAUGCAGCUUUAAAGGAG
Theo 2	CUCCUUUAAAGCGGUUACAAGCCCGCAAAAAUAGCAGAGUAACCCAUACCAGCCGAAAGGCCCUUGGCAGGUUUUAAUGCAGCUUUAAAGGAG
Theo 3	CUCCUUUAAAGCGGUUACAAGCCCGCAAAAAUAGCAGAGUAACUCAUACCAGCCGAAAGGCCCUUGGCAGGUUUUAAUGCAGCUUUAAAGGAG
Theo 4	CUCCUUUAAAGCGGUUACAAGCCCGCAAAAAUAGCAGAGUAAGUCAUACCAGCCGAAAGGCCCUUGGCAGGGUUUAAUGCAGCUUUAAAGGAG
Theo 5	CUCCUUUAAAGCGGUUACAAGCCCGCAAAAAUAGCAGAGUAAACCCAUACCAGCCGAAAGGCCCUUGGCAGGGGUUAAUGCAGCUUUAAAGGAG
Theo 6	CUCCUUUAAAGCGGUUACAAGCCCGCAAAAAUAGCAGAGUAACAACAUACCAGCCGAAAGGCCCUUGGCAGGUUUUAAUGCAGCUUUAAAGGAG
Theo 7	CUCCUUUAAAGCGGUUACAAGCCCGCAAAAAUAGCAGAGUAACGCAUACCAGCCGAAAGGCCCUUGGCAGGCUAUUAAUGCAGCUUUAAAGGAG
Theo 8	CUCCUUUAAAGCGGUUACAAGCCCGCAAUCAUACCAGCCGAAAGGCCCUUGGCAGGUUAUAGCAGAGUAAUGGGAAACCAUUAAUGCAGCUUUAAAGGAG
Theo 9	CUCCUUUAAAGCGGUUACAAGCCCGCAAUUCAUACCAGCCGAAAGGCCCUUGGCAGGGGAAUAGCAGAGUAAUGGGAAACCAUUAAUGCAGCUUUAAAGGAG
TPP 1	CUCCUUUAAAGCGGUUACAAGCCCGCAAAAAUAGCAGAGUAACCUCGGGGUGCCCUUCUGCGUGAAGGCUGAGAAAUACCCGUAUCACCUGAUCUGGAUAAUGCCAGCGUAGGGAAGGUUAAUGCAGCUUUAAAGGAG
TPP 2	CUCCUUUAAAGCGGUUACAAGCCCGCAAAAAUAGCAGAGUAAGCUCGGGGUGCCCUUCUGCGUGAAGGCUGAGAAAUACCCGUAUCACCUGAUCUGGAUAAUGCCAGCGUAGGGAAGCUUAAUGCAGCUUUAAAGGAG
TPP 3	CUCCUUUAAAGCGGUUACAAGCCCGCAAAAAUAGCAGAGUAAUGUCGGGGUGCCCUUCUGCGUGAAGGCUGAGAAAUACCCGUAUCACCUGAUCUGGAUAAUGCCAGCG**G**UAGGGAACCUUAAUGCAGCUUUAAAGGAG
TPP 4	CUCCUUUAAAGCGGUUACAAGCCCGCAAAAAUAGCAGAGUAAGCUCGGGGUGCCCUUCUGCGUGAAGGC**C**UGAGAAAUACCCGUAUCACCUGAUCUGG**G**AUAAUGCCAGCGUAGGGAAGAUUAAUGCAGCUUUAAAGGAG
TPP 5	CUCCUUUAAAGCGGUUACAAGCCCGCAAUUUCGGGGUGCCCUUCUGCGUGAAGGCUGAGAAAUACCCGUAUCACCUGAUCUGGAUAAUGCCAGCGUAGGGAA--AAUAGCAGAGUAAUGGGAAACCAUUAAUGCAGCUUUAAAGGAG
TPP 6	CUCCUUUAAAGCGGUUACAAGCCCGCAAGCUCGGGGUGCCCUUCUGCGUGAAGGCUGAGAAAUACCCGUAUCACCUGAUCUGGAUAAUGCCAGCGUAGGGAAGCAAUAGCAGAGUAAUGGGAAACCAUUAAUGCAGCUUUAAAGGAG
TPP 7	CUCCUUUAAAGCGGUUACAAGCCCGCAACCUCGGGGUGCCC**G**UCUGCGUGAAGGCUGAGAAAUACCCGUAUCACCUGAUCUGGAUAAUGCCAGCGUAGGGAAGGAAUAGCAGAGUAAUGGGAAACCAUUAAUGCAGCUUUAAAGGAG
TPP 8	CUCCUUUAAAGCGGUUACAAGCCCGCAAGGUCGGGGUGCCCUUCUGCGUGAAGGCUGAGAAAUACCCGUAUCACCUGAUCUGGAUAAUGCCAGCGUAGGGAACCAAUAGCAGAGUAAUGGGAAACCAUUAAUGCAGCUUUAAAGGAG
Neo 1	CUCCUUUAAAGCGGUUACAAGCCCGCAAAAAUAGCAGAGUAAGGCGGCAUAGCUUGUCCUUUAAUGGUCCUAUGUCGGGUUAAUGCAGCUUUAAAGGAG
Neo 2	CUCCUUUAAAGCGGUUACAAGCCCGCAAAAAUAGCAGAGUAAAGCGGCAUAGCUUGUCCUUUAAUGGUCCUAUGUCGGGUUAAUGCAGCUUUAAAGGAG

Supplementary Table 4 | Sequences of the theophylline, TPP and neomycin twister-based riboswitches. The SD and the anti-SD sequences are represented in green and red respectively. The theophylline, TPP and neomycin aptamers are highlighted in yellow, cyan and dark blue respectively. The communication modules are represented in magenta. The site that was mutated for the generation of the inactive riboswitches is underlined.
Point mutations in the riboswitch sequences, if present, are represented in bold.

**Controls**			
**eGFP_00**	77,5		
**eGFP_10**	73,7		
**eGFP_01**	73,1		
**eGFP_11**	77,7		
**Background_00**	75,4		
**Background_10**	73,3		
**Background_01**	73,7		
**Background_11**	72		
**Twister active_00**	74,5		
**Twister active_10**	72,4		
**Twister active_01**	74,7		
**Twister active_11**	75,3		
**Twister inactive_00**	70,2		
**Twister inactive_10**	69,8		
**Twister inactive_01**	73,8		
**Twister inactive_11**	71,1		
**Riboswitches**			
**TheoTPP 4.7_00**	77,5	**TheoTPP 7.4_00**	74
**TheoTPP 4.7_10**	73,5	**TheoTPP 7.4_10**	72,6
**TheoTPP 4.7_01**	73	**TheoTPP 7.4_01**	77,2
**TheoTPP 4.7_11**	72,1	**TheoTPP 7.4_11**	72,7
**TheoTPP 4.7 inactive_00**	75,1	**TheoTPP 7.4 inactive_00**	72,6
**TheoTPP 4.7 inactive_10**	77,3	**TheoTPP 7.4 inactive_10**	71,4
**TheoTPP 4.7 inactive_01**	70,3	**TheoTPP 7.4 inactive_01**	72,7
**TheoTPP 4.7 inactive_11**	69,2	**TheoTPP 7.4 inactive_11**	73,2
**TheoTPP 1.4_00**	70,6	**TheoTPP 4.1_00**	81,7
**TheoTPP 1.4_10**	69,7	**TheoTPP 4.1_10**	73,9
**TheoTPP 1.4_01**	79,4	**TheoTPP 4.1_01**	76,2
**TheoTPP 1.4_11**	76,5	**TheoTPP 4.1_11**	73,7
**TheoTPP 1.4 inactive_00**	71,7	**TheoTPP 4.1 inactive_00**	74,4
**TheoTPP 1.4 inactive_10**	69,2	**TheoTPP 4.1 inactive_10**	72,4
**TheoTPP 1.4 inactive_01**	72	**TheoTPP 4.1 inactive_01**	70
**TheoTPP 1.4 inactive_11**	69,7	**TheoTPP 4.1 inactive_11**	72,1
**TheoTPP 4.5_00**	76,3	**TheoTPP 2.2_00**	71,6
**TheoTPP 4.5_10**	71,8	**TheoTPP 2.2_10**	75,8
**TheoTPP 4.5_01**	69,7	**TheoTPP 2.2_01**	75,3
**TheoTPP 4.5_11**	70	**TheoTPP 2.2_11**	70,4
**TheoTPP 4.5 inactive_00**	72,4	**TheoTPP 2.2 inactive_00**	69,4
**TheoTPP 4.5 inactive_10**	72	**TheoTPP 2.2 inactive_10**	70,1
**TheoTPP 4.5 inactive_01**	74,5	**TheoTPP 2.2 inactive_01**	71,5
**TheoTPP 4.5 inactive_11**	72,7	**TheoTPP 2.2 inactive_11**	69,1
**TheoTPP 1.2_00**	76,1		
**TheoTPP 1.2_10**	72,1		
**TheoTPP 1.2_01**	72,5		
**TheoTPP 1.2_11**	70,9		
**TheoTPP 1.2 inactive_00**	68,9		
**TheoTPP 1.2 inactive_10**	73		
**TheoTPP 1.2 inactive_01**	77,3		
**TheoTPP 1.2 inactive_11**	73,2		

Supplementary Table 5 | Percentages of events included in the gated flow cytometry populations of the two-input twister-based riboswitches. The values of the controls are also included.

TheoTPP1.2	CUCCUUUAAAGCGGUUACAAGCCCGCAACCUCGGGGUGCCCUUCUGCGUGAAGGCUGAGAAAUACCCGUAUC<br>ACCUGAUCUGGAUAAUGCCAGCGUAGGGAAGGAAUAGCAGAGUAACCCAUACCAGCCGAAAGGCCCUUGGCA<br>GGGGUUAAUGCAGCUUUAAAGGAG
TheoTPP1.3	CUCCUUUAAAGCGGUUACAAGCCCGCAAUUUCGGGGUGCCCUUCUGCGUGAAGGCUGAGAAAUACCCGUAUC<br>ACCUGAUCUGGAUAAUGCCAGCGUAGGGAA--AAUAGCAGAGUAACCCAUACCAGCCGAAAGGCCCUUGGCA<br>GGGGUUAAUGCAGCUUUAAAGGAG
TheoTPP1.4	CUCCUUUAAAGCGGUUACAAGCCCGCAAACUCGGGGUGCCCUUCUGCGUGAAGGCUGAGAAAUACCCGUAUC<br>ACCUGAUCUGGAUAAUGCCAGCGUAGGGAAGCAAUAGCAGAGUAACCCAUACCAGCCGAAAGGCCCUUGGCA<br>GGGGUUAAUGCAGCUUUAAAGGAG
TheoTPP1.5	CUCCUUUAAAGCGGUUACAAGCCCGCAAGCUCGGGGUGCCC**C**UCUGCGUGAAGGCUGAGAAA**A**UACCCGUAU<br>CACCUGAUCUGGAUAAUGCCAGCGUAGGGAAGUAAUAGCAGAGUAACCCAUACCAGCCGAAAGGCCCUUGGC<br>AGGGGUUAAUGCAGCUUUAAAGGAG
TheoTPP1.6	CUCCUUUAAAGCGGUUACAAGCCCGCAACAUCGGGGUGCCCUUCUGCGUGAAGGCUGAGAAA**A**UACCC**C**GUA<br>UCACCUGAUCUGGAUAAUGCCAGCGUAGGGAAGTAAUAGCAGAGUAACCCAUACCAGCCGAAAGGCCCUUGG<br>CAGGGGUUAAUGCAGCUUUAAAGGAG
TheoTPP2.1	CUCCUUUAAAGCGGUUACAAGCCCGCAAGAUCGGGGUGCCCUUCUGCGUGAAGGCUGAGAAAUACCCGUAUC<br>ACCUGAUCUGGAUAAUGCCAGCGUAGGGAAUUAAUAGCAGAGUAACUCAUACCAGCCGAAAGGCCCUUGGCA<br>GGUUUUAAUGCAGCUUUAAAGGAG
TheoTPP2.2	CUCCUUUAAAGCGGUUACAAGCCCGCAACCUCGGGGUGCCCUUCUGCGUGAAGGCUGAGAAAUACCCGUAUC<br>ACCUGAUCUGGAUAAUGCCAGCGUAGGGAAGGAAUAGCAGAGUAACUCAUACCAGCCGAAAGGCCCUUGGCA<br>GGUUUUAAUGCAGCUUUAAAGGAG
TheoTPP2.3	CUCCUUUAAAGCGGUUACAAGCCCGCAAACUCGGGGUGCCCUUCUGCGUGAAGGCUGAGAAAUACCCGUAUC<br>ACCUGAUCUGGAUAAUGCCAGCGUAGGGAAGGAAUAGCAGAGUAACUCAUACCAGCCGAAAGGCCCUUGGCA<br>GGUUUUAAUGCAGCUUUAAAGGAG
TheoTPP3.1	CUCCUUUAAAGCGGUUACAAGCCCGCAAUACAUACCAGCCGAAAGGCCCUUGGCAGGUAAAUAGCAGAGUAA<br>GCUCGGGGUGCCCUUCUGCGUGAAGGCUGAGAAAUACCCGUAUCACCUGAUCUGGAUAAUGCCAGCGUAGGG<br>AAGCUUAAUGCAGCUUUAAAGGAG
TheoTPP3.2	CUCCUUUAAAGCGGUUACAAGCCCGCAAGUCAUACCAGCCGAAAGGCCCUUGGCAGGGCAAUAGCAGAGUAA<br>GCUCGGGGUGCCCUUCUGCGUGAAGGCUGAGAAAUACCCGUAUCACCUGAUCUGGAUAAUGCCAGCGUAGGG<br>AAGCUUAAUGCAGCUUUAAAGGAG
TheoTPP4.1	CUCCUUUAAAGCGGUUACAAGCCCGCAAAU-A-ACCAGCCGAAAGGCCCUUGGCAGGGUAAUAGCAGAGUAA<br>GCUCGGGGUGCCCUUCUGCGUGAAGG**C**UGAGAAAUACCCGUAUCACCUGAUCUGG**G**AUAAUGCCAGCGUAG<br>GGAAGAUUAAUGCAGCUUUAAAGGAG
TheoTPP4.2	CUCCUUUAAAGCGGUUACAAGCCCGCAAUACAUACCAGCCGAAAGGCCCUUGGCAGGGUAAUAGCAGAGUAA<br>GCUCGGGGUGCCCUUCUGCGUGAAGG**C**UGAGAAAUACCCGUAUCACCUGAUCUGG**G**AUAAUGCCAGCGUAG<br>GGAAGAUUAAUGCAGCUUUAAAGGAG
TheoTPP4.3	CUCCUUUAAAGCGGUUACAAGCCCGCAAGUCAUACCAGCCGAAAGGCCCUUGGCAGGGUAAUAGCAGAGUAA<br>GCUCGGGGUGCCCUUCUGCGUGAAGG**C**UGAGAAAUACCCGUAUCACCUGAUCUGG**G**AUAAUGCCAGCGUAG<br>GGAAGAUUAAUGCAGCUUUAAAGGAG
TheoTPP4.4	CUCCUUUAAAGCGGUUACAAGCCCGCAAGGCAUACCAGCCGAAAGGCCCUUGGCAGGCCAAUAGCAGAGUAA<br>GCUCGGGGUGCCCUUCUGCGUGAAGG**C**UGAGAAAUACCCGUAUCACCUGAUCUGG**G**AUAAUGCCAGCGUAG<br>GGAAGAUUAAUGCAGCUUUAAAGGAG
TheoTPP4.5	CUCCUUUAAAGCGGUUACAAGCCCGCAAAGCAUACCAGCCGAAAGGCCCUUGGCAGGCUAAUAGCAGAGUAA<br>GCUCGGGGUGCCCUUCUGCGUGAAGG**C**UGAGAAAUACCCGUAUCACCUGAUCUGG**G**AUAAUGCCAGCGUAG<br>GGAAGAUUAAUGCAGCUUUAAAGGAG
TheoTPP4.6	CUCCUUUAAAGCGGUUACAAGCCCGCAAGCCAUACCAGCCGAAAGGCCCUUGGCAGGGUAAUAGCAGAGUAA<br>GCUCGGGGUGCCCUUCUGCGUGAAGG**C**UGAGAAAUACCCGUAUCACCUGAUCUGG**G**AUAAUGCCAGCGUAG<br>GGAAGAUUAAUGCAGCUUUAAAGGAG
TheoTPP4.7	CUCCUUUAAAGCGGUUACAAGCCCGCAAAGCAUACCAGCCGAAAGGCCCUUGGCAGGCCAAUAGCAGAGUAA<br>GCUCGGGGUGCCCUUCUGCGUGAAGG**C**UGAGAAAUACCCGUAUCACCUGAUCUGG**G**AUAAUGCCAGCGUAG<br>GGAAGAUUAAUGCAGCUUUAAAGGAG
TheoTPP5.1	CUCCUUUAAAGCGGUUACAAGCCCGCAAUUCAUACCAGCCGAAAGGCCCUUGGCAGGGGAAUAGCAGAGUAA<br>CUUCGGGGUGCCCUUCUGCGUGAAGGCUGAGAAAUACCCGUAUCACCUGAUCUGGAUAAUGCCAGCGUAGGG<br>AA-GUUAAUGCAGCUUUAAAGGAG
TheoTPP5.2	CUCCUUUAAAGCGGUUACAAGCCCGCAAUUCAUACCAGCCGAAAGGCCCUUGGCAGGGGAAUAGCAGAGUAA<br>CCUCGGGGUGCCCUUCUGCGUGAAGGCUGAGAAAUACCCGUAUCACCUGAUCUGGAUAAUGCCAGCGUAGGG<br>AAGGUUAAUGCAGCUUUAAAGGAG
TheoTPP5.3	CUCCUUUAAAGCGGUUACAAGCCCGCAAUUCAUACCAGCCGAAAGGCCCUUGGCAGGGGAAUAGCAGAGUAA<br>GGUCGGGGUGCCCUUCUGCGUGAAGGCUGAGAAAUACCCGUAUCACCUGAUCUGGAUAAUGCCAGCGUAGGG<br>AAGCUUAAUGCAGCUUUAAAGGAG
TheoTPP6.1	CUCCUUUAAAGCGGUUACAAGCCCGCAAUUUCGGGGUGCCCUUCUGCGUGAAGGCUGAGAAAUACCCGUAUC<br>ACCUGAUCUGGAUAAUGCCAGCGUAGGGAA--AAUAGCAGAGUAAAACAUACCAGCCGAAAGGCCCUUGGCA<br>GGUUUUAAUGCAGCUUUAAAGGAG
TheoTPP6.2	CUCCUUUAAAGCGGUUACAAGCCCGCAAUUUCGGGGUGCCCUUCUGCGUGAAGGCUGAGAAAUACCCGUAUC<br>ACCUGAUCUGGAUAAUGCCAGCGUAGGGAA--AAUAGCAGAGUAAGUCAUACCAGCCGAAAGGCCCUUGGCA<br>GGACUUAAUGCAGCUUUAAAGGAG
TheoTPP7.1	CUCCUUUAAAGCGGUUACAAGCCCGCAAGGUCGGGGUGCCCUUCUGCGUGAAGGCUGAGAAAUACCCGUAUC<br>ACCUGAUCUGGAUAAUGCCAGCGUAGGGAACCAAUAGCAGAGUAAGUCAUACCAGCCGAAAGGCCCUUGGCA<br>GGUCUUAAUGCAGCUUUAAAGGAG
TheoTPP7.2	CUCCUUUAAAGCGGUUACAAGCCCGCAAGGUCGGGGUGCCCUUCUGCGUGAAGGCUGAGAAAUACCCGUAUC<br>ACCUGAUCUGGAUAAUGCCAGCGUAGGGAACCAAUAGCAGAGUAAACCAUACCAGCCGAAAGGCCCUUGGCA<br>GGGGUUAAUGCAGCUUUAAAGGAG
TheoTPP7.3	CUCCUUUAAAGCGGUUACAAGCCCGCAAGGUCGGGGUGCCCUUCUGCGUGAAGGCUGAGAAAUACCCGUAUC<br>ACCUGAUCUGGAUAAUGCCAGCGUAGGGAACCAAUAGCAGAGUAAGGCAUACCAGCCGAAAGGCCCUUGGCA<br>GGCUUUAAUGCAGCUUUAAAGGAG
TheoTPP7.4	CUCCUUUAAAGCGGUUACAAGCCCGCAAGGUCGGGGUGCCCUUCUGCGUGAAGGCUGAGAAAUACCCGUAUC

	ACCUGAUCUGGAUAAUGCCAGCGUAGGGAACCAAUAGCAGAGUAAAGCAUACCAGCCGAAAGGCCCUUGGCAGGUUUUAAUGCAGCUUUAAAGGAG
TheoTPP7.5	CUCCUUUAAAGCGGUUACAAGCCCGCAAGGUCGGGGUGCCCUUCUGCGUGAAGGCUGAGAAAUACCCGUAUCACCUGAUCUGGAUAAUGCCAGCGUAGGGAACCAAUAGCAGAGUAAAUCAUACCAGCCGAAAGGCCCUUGGCAGGAUUUAAUGCAGCUUUAAAGGAG
TheoTPP8.1	CUCCUUUAAAGCGGUUACAAGCCCGCAAAAAUAGCAGAGUAACCCAUACCAGCCUCGGGGUGCCCUUCUGCGUGAAGGCUGAGAAAUACCCGUAUCACCUGAUCUGGAUAAUGCCAGCGUAGGGAAGGCCCUUGGCAGGGGUUAAUGCAGCUUUAAAGGAG
TheoTPP8.2	CUCCUUUAAAGCGGUUACAAGCCCGCAAAAAUAGCAGAGUAACCCAUACCAGGAUCGGGGUGCCCUUCUGCGUGAAGGCUGAGAAAUACCCGUAUCACCUGAUCUGGAUAAUGCCAGCGUAGGGAAUCCCCUUGGCAGGGGUUAAUGCAGCUUUAAAGGAG
TheoTPP8.3	CUCCUUUAAAGCGGUUACAAGCCCGCAAAAAUAGCAGAGUAACCCAUACCAGGGUCGGGGUGCCCUUCUGCGUGAAGGCUGAGAAAUACCCGUAUCACCUGAUCUGGAUAAUGCCAGCGUAGGGAAUUCCCUUGGCAGGGGUUAAUGCAGCUUUAAAGGAG
TheoTPP9.1	CUCCUUUAAAGCGGUUACAAGCCCGCAAAAAUAGCAGAGUAACTCAUACCAGGGUCGGGGUGCCCUUCUGCGUGAAGGCUGAGAAAUACCCGUAUCACCUGAUCUGGAUAAUGCCAGCGUAGGGAAUCCCCUUGGCAGGUUUUAAUGCAGCUUUAAAGGAG
TheoTPP9.2	CUCCUUUAAAGCGGUUACAAGCCCGCAAAAAUAGCAGAGUAACTCAUACCAGGCUCGGGGUGCCCUUCUGCGUGAAGGCUGAGAAAUACCCGUAUCACCUGAUCUGGAUAAUGCCAGCGUAGGGAAGCCCCUUGGCAGGUUUUAAUGCAGCUUUAAAGGAG

Supplementary Table 6 | Sequences of the two-input twister-based riboswitches. The SD and the anti-SD sequences are represented in green and red respectively. The theophylline and TPP aptamers are highlighted in yellow and cyan respectively. The connection modules of the theophylline and TPP aptamers are represented in magenta and purple respectively. The site that was mutated for the generation of the inactive riboswitches is underlined.

Name	Sequence	F/R	Description
P1	[Phos]TACAAGCCCGCAAAAATAGCAGAGTAATGGAAGGAGATATACCATGGGCCATCAT	F	Type P1 SD construct
P2	ACCGCTTTATTTCTAAAGCTGCATTAATGGAAGGAGAAAGGGGAATTGTTATCCG	R	Type P1 SD construct
P3	CACGCTTTATTTCTAAAGCTGCATTAATGGAAGGAGAAAGGGGAATTGTTATCCG	R	Type P1 SD construct (inactive Tw together with P1)
P4	[Phos]GAGTAATGGGAAACCATTAATGCAGCTTTAAAGGAGATATACCATGGGCCATCAT	F	Type P3 SD construct
P5	TGCTATTTTTGCGGGCTTGTAACCGCTTTAAAGGAGAAAGGGGAATTGTTATCCG	R	Type P3 SD construct
P6	TGCTATTTTTGCGGGCTTGTACACGCTTTAAAGGAGAAAGGGGAATTGTTATCCG	R	Type P3 SD construct (inactive Tw together with P4)
P7	GAGTAATGGGAAACCATTAATGCAGCTTTAAATTTTGTTTAACTTTAAGAAGGAGATATACC	F	Type P3 5'UTR construct
P8	[Phos]TGCTATTTTTGCGGGCTTGTAACCGCTTTATTTCTAGAGGGGAATTGTTATCCGC	R	Type P3 5'UTR construct
P9	[Phos]CCCGCAAAAATAGCAGAGTAATGG	F	Inactivation Type P3 5'UTR construct
P10	CTTGTACACGCTTTATTTCTAGAGGGG	R	Inactivation Type P3 5'UTR construct
P11	[Phos]GGCCCTTGGCAGGNNTTAATGCAGCTTTAAAGGAGATATACCAT	F	Theo(P1) library 1
P12	TTTCGGCTGGTATGNNTTACTCTGCTATTTTTGCGGGCTT	R	Theo(P1) library 1
P13	TTTCGGCTGGTATGNNNTTACTCTGCTATTTTTGCGGGCTT	R	Theo(P1) library 2
P14	[Phos]GGCCCTTGGCAGGNNNTTAATGCAGCTTTAAAGGAGATATACCAT	F	Theo(P1) library 3
P15	GGCCCTTGGCAGGNNATAGCAGAGTAATGGGAAACCATTAATGC	F	Theo(P5) library 1
P16	TTTCGGCTGGTATGNNTGCGGGCTTGTAACCGCT	R	Theo(P5) library 1
P17	GGCCCTTGGCAGGNNAATAGCAGAGTAATGGGAAACCATTAATGC	F	Theo(P5) library 2
P18	[Phos]TTTCGGCTGGTATGNNTTGCGGGCTTGTAACCGCT	R	Theo(P5) library 2
P19	CGTATCACCTGATCTGGATAATGCCAGCGTAGGGAANNTTAATG	F	TPP(P1) library

	CAGCTTTAAAGGAGATATACCATGG		
P20	[Phos]GGTATTTCTCAGCCTTCACGCAGAAGGGCACCCCGANN TTACTCTGCTATTTTTGCGGGCTTG	R	TPP(P1) library
P21	CGTATCACCTGATCTGGATAATGCCAGCGTAGGGAANNAATAGC AGAGTAATGGGAAACCATTAATGC	F	TPP(P5) library
P22	[Phos]GGTATTTCTCAGCCTTCACGCAGAAGGGCACCCCGANN TTGCGGGCTTGTAACCGCTTTA	R	TPP(P5) library
P23	CGTATCACCTGATCTGGATAATGCCAGCGTAGGGAANNAATAGC AGAGTAACCCATACCAGCCG	F	Two-input library 1
P24	[Phos]GGTATTTCTCAGCCTTCACGCAGAAGGGCACCCCGANN TTGCGGGCTTGTAACCGCT	R	Two-input library 1
P25	CGTATCACCTGATCTGGATAATGCCAGCGTAGGGAANNAATAGC AGAGTAACTCATACCAGCCGAA	F	Two-input library 2 (together with P24)
P26	AGGCCCTTGGCAGGNNAATAGCAGAGTAAGCTCGGGGTGC	F	Two-input library 3 and 4
P27	[Phos]TTCGGCTGGTATGNNTTGCGGGCTTGTAACCGCT	R	Two-input library 3 and 4
P28	[Phos]ACCTGATCTGGATAATGCCAGCGTAGGGAANNTTAATG CAGCTTTAAAGGAGATATACCATGG	F	Two-input library 5
P29	GATACGGGTATTTCTCAGCCTTCACGCAGAAGGGCACCCCGANN TTACTCTGCTATTCCCCTGCCAAGG	R	Two-input library 5
P30	TTTCGGCTGGTATGNNTTACTCTGCTATTTTCCCTACGCTGGC	R	Two-input library 6 (together with P11)
P31	TTTCGGCTGGTATGNNTTACTCTGCTATTGGTTCCCTACGCTG	R	Two-input library 7 (together with P11)
P32	[Phos]CGTATCACCTGATCTGGATAATGCCAGCGTAGGGAANN CCCTTGGCAGGGGTTAATGCAG	F	Two-input library 8
P33	GGTATTTCTCAGCCTTCACGCAGAAGGGCACCCCGANNCTGGTA TGGGTTACTCTGCTATTTTTGCG	R	Two-input library 8
P34	[Phos]CGTATCACCTGATCTGGATAATGCCAGCGTAGGGAANN CCCTTGGCAGGTTTTAATGCAGC	F	Two-input library 9
P35	GGTATTTCTCAGCCTTCACGCAGAAGGGCACCCCGANNCTGGTA TGAGTTACTCTGCTATTTTTGCGG	R	Two-input library 9
P36	CCTTTAAAGCGTGTACAAGCCCGCAA	F	Inactivation *E. Coli* switches
P37	[Phos]AGAAAGGGGAATTGTTATCCGCTC	R	Inactivation *E. Coli* switches
P38	TCTTCACTAGTAAACAAACAAACTCCTTTAAAGCGGTTACAAGC CCGCA	F	Amplification Tw from pET16 and insertion NotI and SpeI sites
P39	TCTTCACTAGTTCTTCTCTTCGCGGCCGCTTTTTCTTTTTCTCC TTTAAAGCTGCATTAATGGTTTCC	R	Amplification Tw from pET16 and insertion NotI and SpeI sites
P40	TCTTCACTAGTAAACAAACAAACTCCTTTAAAGCGTGTACAAGC CCGCA	F	Amplification inactive Tw from pET16 and insertion NotI and SpeI sites (together with P39)
P41	[Phos]CTTTAATGGTCCTATGTCGNNTTAATGCAGCTTTAAAG GAGAAAAAGAAAA	F	Neo(P1) library
P42	GACAAGCTATGCCGNNTTACTCTGCTATTTTTGCGGGCTTG	R	Neo(P1) library
P43	CCCGCAAAAATAGCAGAGTAA	F	Inactivation Neo switches on shuttle vector
P44	[Phos]CTTGTACACGCTTTAAAGGAGTTTG	R	Inactivation Neo switches on shuttle vector
P45	AGAATACATATGAAATTTGGAAACTTTTTGCTTACATACCAA	F	Amplification of luxAluxB and insertion NdeI site
P46	AGAATAGGATCCCTATTAGGTATATTCCATGTGGTACTTCTTAA TATTATCATC	R	Amplification of luxAluxB and insertion BamHI site

Supplementary Table 7 | List of primers employed for the generation of the plasmids. 5'-phosphorylated primers are indicated with [Phos].

Supplementary Note 1: Twister ribozyme as expression platform in artificial riboswitches regulating translation initiation in *E. coli*

In order to prove that in *E.coli* our artificial twister-based riboswitches exert their gene expression control activity through the sequestration of the Shine-Dalgarno sequence (SD), we compared the mRNA levels of five different constructs (**Supplementary Fig. 2a-c**). *ssrA* was used as an internal standard. The real time qPCR data show that the mRNA levels of the five constructs are comparable (**Supplementary Fig. 2d**), indicating that the cleavage activity in the 5'-UTR of the reporter gene does not have in itself an effect on the levels of eGFP expression. To test the H_0 hypothesis that mRNA levels do not differ significantly in the five constructs we performed a Kruskal-Wallis test (with k = 5 samples and n_i = 3 number of observations per sample). The computed H value resulted to be equal to 5 with P = 0.2873, hence the H_0 hypothesis was accepted.

As already mentioned in the main text, when the SD sequence is masked and inserted into stem P3 of the catalytic motif, a strong difference in the expression levels of the eGFP is observed between the active and the inactive constructs. The qPCR data show that in these two constructs the mRNA levels do not differ significantly (**Supplementary Fig. 2d**), indicating that the drastic decrease of the eGFP expression is only due to the permanent masking of the SD sequence. Taken together these data indicate that in our artificial system, the control of the eGFP expression is not dependent on the cleavage of the 5'-UTR of the gene and on the stability of the mRNA, but it is exerted exclusively at the level of translation initiation by sequestering the SD sequence.

Supplementary Note 2: Theophylline- and TPP-dependent artificial riboswitches based on the twister ribozyme

The catalytic activity of the twister ribozyme can be modulated by connecting a sensor domain to the catalytic moiety. Two possible regulation mechanisms can be proposed in *E. coli*. In case of on-switches the catalytic domain is activated in the presence of the ligand, resulting in the liberation of the SD sequence and the expression of the eGFP (**Supplementary Fig. 3a**). In case of off-switches the ligand acts as an inhibitor of the catalytic activity of the ribozyme, leading to the sequestration of the SD sequence and the inhibition of the eGFP expression (**Supplementary Fig. 3b**).

The twister motif offers several attachment sites to introduce ligand-dependent aptamer sequences. The stem structure P1 is present in all identified twister motives and consists of a non-conserved sequence

of variable length[1]. In P1-type twister, the helix P1 connects the ribozyme to the mRNA. The crystal structure of the Osa-1-4 motif showed that the nucleotides of helix P1 are not directly involved in the formation of the active site[2]. Although the stem P1 was found in all twister motives, recently an artificial minimal version of the *env22* type P3 motif was reported that lacks the stem P1[3]. The close proximity of the stem P1 to the catalytic core and his low conservation make this site an ideal candidate for the attachment of a ligand sensor domain.

The stem P5 is only found in 1% of the P1-type twister ribozymes[1]. It is located in a loop which connects stem P4 to stem P2. The nucleotides of this loop are not conserved and are not involved in the formation of the active site. In P5-type twister, the position P5 is the site where the ribozyme is connected to the mRNA. Although the stem P5 is generally variable in length and composition, the crystallographic data suggest that its presence could have a critical role in the maintenance of the overall structure in some twister ribozymes[2]. None of the naturally identified P3-type twister ribozymes, including the one employed in this work (env-9), comprises a stem in position P5.

Our screening strategy led to the development of a series of theophylline- and TPP-dependent artificial riboswitches. The **Supplementary Figs. 4** and **5** show the selected communication modules and the respective switch performances of active and inactivated twister riboswitches. When the catalytic domain is specifically inactivated, the eGFP expression is completely switched off due to the permanent sequestration of the SD sequence.

For theophylline P1 aptazymes we tested three different types of communication modules: one symmetric design containing four randomized nucleotides and two asymmetric containing five randomized nucleotides (**Supplementary Fig. 4a**). Our data show that the number of nucleotides in the communication module can be easily optimized in order to improve the switching activity in response to ligand addition. The theophylline-dependent riboswitches presenting an asymmetric communication module display a higher activity compared to the ones presenting a symmetric communication module (**Supplementary Fig. 4c,d**).

In the P5 theophylline aptazymes, symmetric communication modules of four randomized nucleotides were inserted by replacing a variable number of residues in the accommodating loop (**Supplementary Fig. 4a**). The screening of the different libraries resulted in the isolation of only two weak switches (more than 2-fold inactivation) with a different number of flanking adenines in the communication modules (**Supplementary Fig. 4b-d**). The presence of at least one adenine residue in the flanking sequences seems to be important for the development of switches with an aptamer domain in position P5. The TPP P1 and P5 libraries were designed using symmetric communication modules of four randomized nucleotides (**Supplementary Fig.**

5a). Interestingly the employment of the naturally occurring TPP aptamer domain resulted in twister-based riboswitches with a higher biological activity compared to theophylline riboswitches in both P1- and P5-based constructs (**Supplementary Fig. 5c,d**). All the one-input riboswitches were further characterized by flow cytometry (FC) (**Supplementary Fig. 6-8**). The gating procedure is illustrated in **Supplementary Fig. 6** for the eGFP positive control populations in three different culture conditions (no ligand, 2.5 mM theophylline and 1 mM thiamine – See **Methods** section for details). The same gate was applied on all samples. The percentages of events (cells) included in the gated population for each sample (controls and riboswitches) are shown in **Supplementary Table 1**. Quantitative comparisons of the histograms recorded in the presence and in the absence of ligand were performed for each sample (controls and riboswitches). T(X) values were calculated and are shown in **Supplementary Fig. 6-8**. All the T(X) values of the one-input switches were shown to be significantly larger than the T(X) values calculated for the four control samples (**Supplementary Fig. 6-8**)[4]. Hence all the analyzed one-input riboswitches showed high to moderate switching performances at the level of single cell.

Supplementary Note 3: Theophylline and TPP aptazyme kinetics measured by *in vitro* cleavage assays

Four ligand-dependent twister ribozymes were further characterized *in vitro*. In particular we determined cleavage kinetics of the *in vitro* transcribed aptazymes in a monomolecular construct. Selected aptazymes were incubated in the presence and in the absence of the respective ligand and aliquots of the reaction mix were collected at different time points. The env-9 twister motif was already kinetically characterized by Roth *et al.* using a bimolecular construct [1]. Under standard reaction conditions the k_{obs} of this construct approached 10 min^{-1}. The k_{obs} under physiological conditions was estimated to approach 1,000 min^{-1}. Due to the high cleavage activity of the ribozyme it was not possible to determine an accurate rate constant of the env-9 motif in the absence and in the presence of the theophylline and TPP.
Supplementary Fig.9 shows the kinetics of Theo1, Theo3, TPP2 and TPP4 riboswitch motives in the absence and in the presence of the respective ligand. In the left column the full timecourse is shown whereas on the right only the first time points are represented. Here it is possible to see qualitatively the effect of the ligand addition on the rate of reaction. In the off-switches we observed a decrease in the rate of reaction whereas in the on-switches we saw an increase in the rate of reaction upon ligand addition. The

magnitude of activation/inactivation *in vitro* seems to be more pronounced in Theo3 and TPP2 (**Supplementary Fig. 9b,c**) and less in the Theo1 and TPP4 (**Supplementary Fig. 9a,d**). The switch Theo1 resulted to be the fastest aptazyme of the ones we tested. However for a better determination of the kinetic parameters it would be necessary to have more time points in the first 15 seconds of the reaction, which resulted to be not possible with our manual method. Similarly more time points in the first 15 seconds of reaction are needed for a better kinetic characterization of the TPP2 switch in the absence of the ligand.

The activity of the twister ribozyme was shown to be strongly dependent on the Mg^{2+} concentration and on the pH of the solution[1]. Working in suboptimal reaction conditions (lower Mg^{2+} concentration and lower pH) could help to better visualize the difference between on- and off-state of the riboswitches.

The kinetic traces were fitted using two different equations:

$$y = A \cdot e^{-x/t} + y_0 \qquad (1)$$

$$y = A_1 \cdot e^{-x/t1} + A_2 \cdot e^{-x/t2} + y_0 \qquad (2)$$

The coefficients of determination (R^2) of the different fittings were calculated and compared. According to Winkler et al. the GlmS ribozyme in 1mM Mg^{2+} with a ligand (GlcN6P) concentration of 200 µM the k_{obs} is ~ 0.01 min^{-1}. The k_{obs} of the GlmS ribozyme shows a log-linear dependency on the ligand concentration, with a minimum k_{obs} value of 0.003 min^{-1} (200 nM GlcN6P) and a maximum value of 3 min^{-1} (around 1 mM GlcN6P). In comparison, our aptamer-controlled ribozymes show k_{obs} values higher or comparable to the one of the GlmS ribozyme in similar conditions if a single exponential fitting is used[5]. Interestingly the mono-exponential fitting (1) resulted to be the best for kinetic traces of the aptazyme in the absence of the ligand (**Supplementary Table 2**). The bi-exponential fitting (2) resulted to be the best for all the switches when the ligand is present in the reaction mix (**Supplementary Table 3**).

This biphasic behavior can be explained by the existence of a ligand-dependent kinetic process, taking place in addition to the cleavage reaction:

- Binding of the ligand to the aptamer domain. This event is supposed to be very fast and hence not likely to be seen using manual methods of kinetic determination.
- Conformational change of the ribozyme upon ligand binding.
- Contemporaneous cleavage reactions of the aptazyme-ligand complex and of the free aptazyme. In this case at least one of the rate constants of the bi-exponential equation should be comparable to rate constant calculated for the reaction in the absence of ligand (here fitted with a mono-exponential equation).

In summary, although the difference between the on- and the off-state in our kinetic traces are not always pronounced, our *in vitro* assay seems to support the mechanism of genetic regulation proposed and described in the **Supplementary Fig. 3**.

The analyzed aptazymes show better performances when tested *in vivo* then *in vitro*. It is, however, important to mention that the actual protein expression level in the cell is determined by a complex chain of events such as transcription, RNA self-cleavage, RNA degradation and protein synthesis[6]. The *in vitro* conditions might not reproduce the cellular environment accurately. First of all the aptazyme motif is just a small part of a much longer mRNA in the cell. Co-transcriptional folding and cellular molecular crowding were shown to play an important role in the determination of the performances of artificial riboswitches that regulate gene expression at the translational level[7]. Moreover the activity of an artificial riboswitch *in vivo* can be strongly influenced by factors like divalent ion concentration, pH, temperature and interactions with other species present in the cytoplasm. All these factors can have a strong influence not only on the cleavage rate constant of the ribozyme and the binding of the inducer by the aptamer domain, but also can play a role in the formation of alternative cleavage inactive structures, which in turn affect the total fraction of cleaved RNA. In contrast, even very pronounced ligand-induced changes of cleavage activity do not necessarily translate into changes of gene expression, as demonstrated by Breaker and coworkers[8].

Supplementary Note 4: Neomycin-dependent artificial riboswitches based on the twister ribozyme

In order to develop artificial riboswitches based on twister in yeast, an *in vivo* screening approach was used. The library of twister-based aptazymes was inserted in the 3'UTR of the gene encoding for the GAL4 transcription factor. Here, the removal of the poly(A) tail upon cleavage reduce the stability of the mRNA (**Supplementary Figure 4**). The screening strategy is based on the detection of the activity of the β-galactosidase encoded by the *lacZ* gene, whose transcription is directly activated by GAL4. The library was generated attaching the neomycin aptamer to the stem P1 of the twister using a four randomized nucleotides communication module (**Supplementary Fig. 11a**). The library was screened directly in yeast for differential expression of β-galactosidase in the presence and in the absence of neomycin in yeast culture medium. The screening resulted in the isolation of two effective off-switches that present purine nucleotide-rich communication modules (**Fig. 2** and **Supplementary Fig. 11a-c**). Interestingly no on-switch was isolated during the screening. Catalytically inactive mutants of the artificial riboswitches do not show off-switch behavior indicating that the cleavage is essential for the aptazyme-mediated gene expression regulation

(**Supplementary Fig. 11b-c**). An analogous library screening in position P5 did not result in any effective neomycin riboswitch.

Supplementary Note 5: Two-input twister-based riboswitches

For the generation of twister-based two-input riboswitches we took advantage of the fact that the twister ribozyme presents two possible sites for the attachment of aptamer domains (P1 and P5). The rational design approach previously employed by other groups[9, 10] was demonstrated to be a valid method for the production of some specific Boolean logic gates (OR, AND, NOR and NAND), however it seems to be restricted regarding the generation of a broad range of Boolean logic gates using a single ribozyme as expression platform.

For these reasons we decided to use a screening approach for the generation of two-input Boolean logic gates based on twister. In our screening approach we started from one of the one-input switches previously characterized (**Supplementary Figs. 4b** and **5b**), and we inserted the second aptamer in the other position connecting it with a randomized symmetric communication module of four nucleotides (**Supplementary Fig. 12**). We screened in total seven libraries:

- Library 1: Starting from Theo1 switch (theophylline aptamer in P1 off) – screening TPP aptamer in P5
- Library 2: Starting from Theo3 switch (theophylline aptamer in P1 on) – screening TPP aptamer in P5
- Library 3: Starting from TPP2 switch (TPP aptamer in P1 off) – screening theophylline aptamer in P5
- Library 4: Starting from TPP4 switch (TPP aptamer in P1 on) – screening theophylline aptamer in P5
- Library 5: Starting from Theo19 switch (theophylline aptamer in P5 off) – screening TPP aptamer in P1
- Library 6: Starting from TPP5 switch (TPP aptamer in P5 off) – screening theophylline aptamer in P1
- Library 7: Starting from TPP7 switch (TPP aptamer in P5 off) – screening theophylline aptamer in P1

Although we observed a general decrease in the levels of eGFP expression when two sensor domains are present on the same catalytic core, the screening approach resulted to be more flexible compared to the rational design approach employed previously with different ribozyme scaffolds[9, 10].

We were able to identify a broad range of Boolean logic gates (more than 20 switches) that differed in the reactivity and performances (**Supplementary Figs 13-19**). Depending on where the threshold is set, the twister-based two-input riboswitches can be described as AND, NAND, OR, NOR, theophylline ANDNOT TPP and TPP ANDNOT theophylline. Occasionally some switches that show different behavior present only small differences in the sequence. For example TheoTPP2.2 (ANDNOT1) and TheoTPP2.3 (Theo ON) differ

for only one nucleotide in one of connection modules (**Supplementary Fig. 14c,d**). In a similar way TheoTPP4.1 and TheoTPP4.6 are converted into TheoTPP4.3 by single A to G and C to U mutations respectively with their reactivity changing from ANDNOT2 and AND to TPP ON-buffer (**Supplementary Fig. 16b,d,g**). Some of the selected twister-based switches show dependency on only one of the two ligands even if they carry two different aptamer domains. These riboswitches can be described as Boolean buffer or inverter gates.

Some of the two-input riboswitches were characterized also by the FC (**Fig. 3** and **Supplementary Fig. 20-21**). The gate shown in **Supplementary Fig. 6** was applied to all two-input riboswitches samples. The percentages of events included in the gated population for each sample are shown in **Supplementary Table 5**. T(X) values were calculated and are shown in **Supplementary Fig. 20-21**. All the relevant T(X) values of the two-input switches were shown to be significantly larger than the T(X) values calculated for the four control samples (**Supplementary Fig. 20**). Some of the two-input riboswitches showed a less pronounced switching behavior when compared to the one-input riboswitches, however the patterns of response to the presence of the ligand in the culture medium remain clearly recognizable also in the FC diagrams.

In some case FC cytometry allowed a finer characterization of some two-input riboswitches. This is the case of the ANDNOT2 logic gates in the **Supplementary Fig. 21**. Here in presence of theophylline in the culture medium, the clones shows a significantly decrease in eGFP expression. Setting the threshold opportunely this expression pattern can be described as an ORNOT logic gate (the table of truth is also represented in **Supplementary Fig. 21**).

Supplementary Note 6: Validation of selected one-input and two-input riboswitches using bacterial luciferase as a reporter gene.

We further tested some of the one- and two-input riboswitches with in a bioluminescence assay, exchanging the eGFP open reading frame with the *luxA* and *luxB* genes encoding for a bacterial luciferase (luxAB). The main benefit of the bioluminescence assay is the high signal to noise ratio due to the absence of background luminescence in the cells not expressing luxAB. Although the use eGFP as a reporter gene presents the advantages of being reliable and fast, the influence of the background fluorescence could be relevant in the low eGFP-expressing clones. The chemoluminescence time courses of one minute upon the addition of the substrate (decanal) are shown in **Supplementay Fig. 22**. In order to avoid the effect due to

the variability in the absolute values of luminescence between the biological replicates, the data are reported as relative to the maximum chemoluminescence recorded for each replicate.

The data of chemoluminescence reproduce the results observed using eGFP as a reporter gene for both the one- and the two-input riboswitches. TheoTPP1.4, which can be described as a NAND logic gate in the fluorescence assays, shows a clear NOR behavior in the bioluminescence assay. The use of multiple reporter genes seems to be a critical point for an accurate characterization of the two-input riboswitches, especially if the levels of expression of the target gene are low and are finely tuned by the artificial RNA device.

Supplementary Note 7: Two-input riboswitches generated using aptamer domains connected in series

The reduced expression levels of the two-input riboswitches presented in **Fig. 3** and **Supplementary Fig. 13-19** are probably due to the presence on the same catalytic core of two different sensor domains in the position P1 and P5. This on one hand allows a ligand-dependent control of the cleavage activity of the twister, on the other hand strongly decreases the catalytic activity of the ribozyme. An alternative strategy is to connect the two sensor domain in one site of the ribozyme utilizing an in series design[11].

Starting from Theo1 (OFF) and Theo3 (ON) switches we generated two libraries connecting the TPP aptamer to the terminal stem-loop of the theophylline aptamer. The nucleotides involved in the binding of theopylline were not modified[12]. The TPP aptamer was connected to the theophylline aptamer using a communication module of four randomized nucleotides (**Supplementary Fig. 23**). The two clone libraries were screened for differential expression of eGFP in four different conditions (no ligand, 2.5 mM theophylline, 1 mM thiamin, 2.5 mM theophylline and 1 mM thiamin).

The screening resulted in the isolation of a few two-input riboswitches which present higher eGFP expression levels compared to the two-input riboswitches described previously in this work. However this design seems to be less flexible in regard of variety of switching behavior observed (**Supplementary Fig. 24-25**). It is interesting to notice how the TPP aptamer can still strongly influence the activity of the switch despite his location far away from the catalytic core of the twister motif.

Supplementary References:

1. Roth, A. et al. A widespread self-cleaving ribozyme class is revealed by bioinformatics. *Nat Chem Biol* **10**, 56-60 (2014).
2. Liu, Y., Wilson, T.J., McPhee, S.A. & Lilley, D.M. Crystal structure and mechanistic investigation of the twister ribozyme. *Nat Chem Biol* (2014).
3. Kosutic, M. et al. A Mini-Twister Variant and Impact of Residues/Cations on the Phosphodiester Cleavage of this Ribozyme Class. *Angew Chem Int Ed Engl* (2015).
4. Roederer, M., Treister, A., Moore, W. & Herzenberg, L.A. Probability binning comparison: a metric for quantitating univariate distribution differences. *Cytometry* **45**, 37-46 (2001).
5. Winkler, W.C., Nahvi, A., Roth, A., Collins, J.A. & Breaker, R.R. Control of gene expression by a natural metabolite-responsive ribozyme. *Nature* **428**, 281-286 (2004).
6. Kobori, S., Ichihashi, N., Kazuta, Y., Matsuura, T. & Yomo, T. Kinetic analysis of aptazyme-regulated gene expression in a cell-free translation system: modeling of ligand-dependent and -independent expression. *RNA* **18**, 1458-1465 (2012).
7. Espah Borujeni, A., Mishler, D.M., Wang, J., Huso, W. & Salis, H.M. Automated physics-based design of synthetic riboswitches from diverse RNA aptamers. *Nucleic Acids Res* **44**, 1-13 (2016).
8. Link, K.H. et al. Engineering high-speed allosteric hammerhead ribozymes. *Biol Chem* **388**, 779-786 (2007).
9. Nomura, Y., Zhou, L., Miu, A. & Yokobayashi, Y. Controlling mammalian gene expression by allosteric hepatitis delta virus ribozymes. *ACS Synth Biol* **2**, 684-689 (2013).
10. Win, M.N. & Smolke, C.D. Higher-order cellular information processing with synthetic RNA devices. *Science* **322**, 456-460 (2008).
11. Jose, A.M., Soukup, G.A. & Breaker, R.R. Cooperative binding of effectors by an allosteric ribozyme. *Nucleic Acids Res* **29**, 1631-1637 (2001).
12. Zimmermann, G.R., Wick, C.L., Shields, T.P., Jenison, R.D. & Pardi, A. Molecular interactions and metal binding in the theophylline-binding core of an RNA aptamer. *RNA* **6**, 659-667 (2000).
